# DNA Methylation Alterations in Patients with Moderate-to-Severe Thyroid Eye Disease Treated with Intravenous Glucocorticoids

**DOI:** 10.3390/genes17101181

**Published:** 2026-09-24

**Authors:** Mikołaj Radziszewski, Piotr Gasperowicz, Aleksander Kuś, Grażyna Kostrzewa, Rafał Płoski, Tomasz Bednarczuk

**Affiliations:** 1 Department of Internal Medicine and Endocrinology, Medical University of Warsaw, 02-097 Warsaw, Poland; aleksander.kus@wum.edu.pl (A.K.); tomasz.bednarczuk@wum.edu.pl (T.B.); 2 Department of Medical Genetics, Centre for Biostructure, Medical University of Warsaw, 02-106 Warsaw, Poland; piotr.gasperowicz@wum.edu.pl (P.G.); grazyna.kostrzewa@wum.edu.pl (G.K.); rafal.ploski@wum.edu.pl (R.P.)

**Keywords:** autoimmunity, Graves’ disease, thyroid eye disease, DNA methylation, RRBS, glucocorticoids

## Abstract

**Background/Objectives:** Genome-wide epigenetic alterations in thyroid eye disease (TED) remain insufficiently characterized, particularly in the context of intravenous glucocorticoid (ivGC) therapy. We aimed to characterize genome-wide DNA methylation (DNAm) alterations in patients with active, moderate-to-severe TED and assess differences in DNAm patterns observed following ivGC therapy and in relation to clinical response. Methods: We enrolled 24 euthyroid patients with moderate-to-severe TED and 7 healthy controls (HC). Peripheral whole-blood DNAm profiling was performed using reduced representation bisulfite sequencing before (TED-P1) and 12 weeks after ivGC therapy (TED-P2), with both time points compared with the same HC reference group. Within-patient DNAm changes were additionally explored at the individual level. Gene set enrichment (GSE) analysis investigated the biological context of identified methylation alterations. Results: Genome-wide and site-specific DNAm differences were identified between TED patients and HC, with a marked predominance of hypomethylation. Principal component analysis demonstrated separation of TED patients from HC, with differences in clustering patterns observed after treatment. GSE identified enrichment of pathways related to immune regulation, cell-cycle control and, among other categories, visual system development. Sensitivity analyses addressing smoking status and age supported the persistence of the predominant hypomethylation pattern. Exploratory analyses suggested differences in post-treatment DNAm patterns according to cumulative ivGC exposure, whereas no consistent DNAm signature associated with clinical treatment response was identified in this cohort. Conclusions: Patients with moderate-to-severe TED exhibited genome-wide and site-specific DNAm alterations characterized predominantly by hypomethylation and functionally relevant pathway enrichment. These findings require validation in larger, independent cohorts.

## 1. Introduction

Graves’ disease (GD) is an autoimmune thyroid disease (AITD) with a multifactorial pathogenesis involving genetic, environmental, and endogenous factors [1,2]. The pathogenesis of GD involves complex interactions between lymphocytes and thyrotropin receptor autoantibodies (TRAb), which activate the thyroid-stimulating hormone receptor (TSHR) [1,3]. The effect of this is hyperthyroidism, but the condition also manifests in skin and orbital soft tissue, leading, respectively, to dermopathy and thyroid eye disease (TED) [4].

TED is the main extra-thyroidal manifestation of GD and is strongly associated with age, high TRAb levels and cigarette smoking [5]. TED exhibits considerable variability in disease activity and severity and may substantially impair quality of life or, in severe cases, threaten vision [6,7,8,9]. According to the European Group on Graves’ Orbitopathy (EUGOGO), intravenous glucocorticoids (ivGCs) remain a key treatment option for active, moderate-to-severe TED, although therapeutic response remains variable [8]. Given the heterogeneous clinical course of TED and the incompletely understood mechanisms underlying different GD phenotypes, the identification of novel biomarkers, including epigenetic markers, remains of considerable interest [10,11,12].

Among epigenetic mechanisms, DNA methylation (DNAm) has attracted particular interest because of its role in the regulation of gene expression and its potential responsiveness to environmental factors [13,14]. DNAm is mediated by DNA methyltransferases (DNMTs), which catalyze the addition of a methyl group to cytosine (C), generating 5-methylcytosine (5-MC) and commonly found in stretches of cytosine–phosphate–guanine (CpG) dinucleotides [13,15]. Notably, CpGs occur in dense concentrations in the form of CpG islands (CGIs) located predominantly in gene-promoter regions [15]. Considering the multitude of techniques available for DNAm assessment, reduced representation bisulfite sequencing (RRBS) enables quantitative, genome-wide analysis with CpG methylation profiling within CpG-enriched regions, providing an efficient compromise between resolution, coverage, biological relevance and cost [16,17,18].

In fact, evidence supporting DNAm alterations in autoimmune disorders, including GD, is gaining importance [19,20,21,22]. However, most studies have focused on site-specific DNAm alterations, and genome-wide methylation profiling using next-generation sequencing remains limited [23,24,25]. Moreover, although aberrant DNAm has been reported in GD, little is known about treatment-associated longitudinal epigenetic changes or the genome-wide methylation landscape of TED [26]. In turn, GCs have been found to modify DNAm patterns, but also to influence immune-cell functions in GD [27,28,29,30].

Considering the above, the primary objective of this study was to characterize genome-wide and site-specific DNAm alterations in peripheral whole blood from patients with active, moderate-to-severe TED using RRBS, with particular emphasis on differences between patients at baseline and healthy controls. The secondary objective was to investigate longitudinal DNAm changes associated with standard ivGC therapy through within-patient comparisons at the individual-sample level and by evaluating post-treatment methylation patterns in relation to the healthy-control reference.

## 2. Materials and Methods

### 2.1. Subject Characteristics

In this study, we enrolled consecutive adult patients (≥18 years) with GD and active, moderate-to-severe TED, diagnosed according to European Thyroid Association and EUGOGO guidelines [8,31]. Only euthyroid patients at the time of inclusion were eligible. Disease activity was assessed using the 7-point Clinical Activity Score (CAS), with active TED defined as CAS ≥ 3/7. Patients who had received systemic GC within 6 months prior to initiation of ivGC therapy were excluded.

Healthy adult controls (HC) with no history of thyroid disease, autoimmune disorders, smoking, prior GC treatment or pregnancy were also recruited. The study protocol was approved by the Ethics Committee of the Medical University of Warsaw (KB/151/2019) and conducted in accordance with the Declaration of Helsinki. Written informed consent was obtained from all participants.

### 2.2. Study Design

This prospective study combined two case–control comparisons (TED versus healthy controls at baseline and after treatment) with a longitudinal within-patient assessment of DNAm changes following intravenous glucocorticoid therapy. Clinical data were collected for all patients, including (i) TED characteristics according to EUGOGO criteria, (ii) previous GD treatment, (iii) smoking status, classified as ever-smokers (current or former smokers) or never-smokers, and (iv) family history of AITD. Patients underwent clinical and laboratory evaluation at two time points: before treatment initiation (TED-P1) and after completion of ivGC therapy (TED-P2), as shown in Figure 1 and Table 1. Laboratory assessment included thyroid-related immunoassays and genome-wide DNAm analysis using RRBS [32].

According to EUGOGO recommendations, patients received weekly ivMP pulses, consisting of six consecutive infusions of 0.5 g or 0.75 g followed by six infusions of 0.25 or 0.5 g, respectively, with a cumulative dose adjusted to disease severity [8]. In selected cases, combined orbital radiotherapy (2000 cGy/week) was administered. Clinical evaluation was based on the more affected eye.

Treatment response at TED-P2 was defined using a modified Composite Index, as previously described [8,33]. Improvement required at least two of the following in the same eye, without deterioration in the fellow eye: (i) at least a 3 mm reduction in the palpebral aperture, (ii) a reduction in exophthalmos by at least 2 mm, (iii) improved motility in any direction, or an improvement in diplopia (based on Gorman score, from 0 to 3), and (iv) a decrease in CAS of at least 2 points.

### 2.3. Laboratory Methods: Immunoassay, DNA Libraries and Sequencing

Serum TSH, free thyroxine and triiodothyronine (fT4 and fT3), as well as TRAb levels were measured in TED patients at the two time points (TED-P1 and TED-P2) using electrochemiluminescence immunoassays (Roche Diagnostics, Elecsys, Olathe, KS, USA, Cobas analyzer). In HC, only serum TSH was assessed. Peripheral venous blood was collected into EDTA tubes from both TED patients (2 samplings, before and after ivGC therapy: TED-P1 and TED-P2) and HC (1 sampling). Samples were stored at −20 °C. DNA was isolated and purified using the Genomic Maxwell RSC Whole Blood DNA Kit (Promega Corporation, Madison, WI, USA) and Maxwell Instrument according to the manufacturer’s protocol. Sample quality and DNA concentration quantification were verified using a Qubit 3.0 fluorometer (Thermo Fisher Scientific, Invitrogen, Waltham, MA, USA).

DNA libraries were prepared using the NEXTflex Bisulfite Seq Library Prep kit (PerkinElmer, Inc., Austin, TX, USA). Briefly, genomic DNA was digested with restriction enzyme MsPI (New England Biolabs, Inc., Ipswich, MA, USA; CCGG sites), followed by end-repair, adenylation, adapter ligation with indexed adapters and size-selection using magnetic bead-based purification (Vazyme Biotech Co., Ltd., Nanjing, China). Bisulfite conversion was performed using EZ DNA Methylation-Lightning Kit (Zymo Research Corp., Irvine, CA, USA), followed by polymerase chain reaction (PCR) amplification, yielding fragments of approximately 350–400 bp. Library quality and concentration were confirmed using Qubit 3.0 device (Thermo Fisher Scientific, Invitrogen, Waltham, MA, USA), as well as an Agilent 2100 Bioanalyzer (Agilent Technologies, Santa Clara, CA, USA).

The NGS technique was performed on an Illumina NovaSeq 6000 System (Illumina, Inc., San Diego, CA, USA), using paired-end sequencing (2 × 150 bp reads), generating high-resolution methylation data with depth of 50 million reads per sample.

### 2.4. Results Processing: Raw-Data Differential Analysis of DNAm, Visualization and Statistical Approach

DNAm alterations were evaluated at both genome-wide and site-specific levels. Analyses included identification of differentially methylated cytosines (DMCs) and differentially methylated regions (DMRs) based on CpG annotation. The analytical strategy reflected the predefined study objectives. First, baseline DNAm patterns in TED-P1 were compared with HC to characterize TED-associated methylation differences. Second, post-treatment DNAm patterns were evaluated by comparing TED-P2 samples with the same HC reference group, while longitudinal within-patient DNAm changes were explored separately at the individual-sample level. Exploratory analyses subsequently evaluated DNAm patterns according to clinical treatment response and cumulative ivGC exposure. Functional enrichment analyses (Gene Set Enrichment; GSE) were used to investigate the biological context of the identified methylation alterations. Finally, additional sensitivity analyses assessed the potential influence of selected confounding factors, including age, smoking status, and longitudinal changes in routinely measured peripheral blood-cell composition.

Statistical and bioinformatic analyses were performed using R version 4.4.0, including the methylKit package (v1.30.0) for differential methylation analysis, and SAS version 9.4 (SAS Institute Inc., Cary, NC, USA). Raw FASTQ files underwent initial quality control, followed by adapter trimming using Cutadapt. Reads were subsequently aligned to the hg19 reference genome using Bismark, generating Binary Alignment Map (BAM) files for downstream methylation analysis. Further data processing for the statistical analyses was performed using our in-house pipelines, which are publicly available online at: https://github.com/pgasperowicz/MethApp (accessed on 21 September 2026) [34]. Sex chromosomes were eliminated from analysis given potentially confounding sex-specific methylation patterns [35].

We investigated genomic and CpG-related annotations, showing the overall distribution of DMCs obtained across predefined features using the ChIPseeker package (v1.40.0), in regard to: (i) promoters (defined as within the range −1500 to +500 bp from transcription start sites, TSS), (ii) introns (non-coding regions between exons), (iii) exons (protein-coding regions), (iv) “downstream” regions (≤300 bp from where the gene ends; 3′ flanking region), (v) intergenic regions (between genes), (vi) 3′ untranslated regions (after the coding sequence ends; 3′UTR) and (vii) 5′ untranslated regions (before the coding sequence starts; 5′UTR) [34]. We also analyzed CpG context distribution, including: (i) CpG Islands (CGI), (ii) CpG Shores (CGS; ±2000 bp flanking a CGI), (iii) CpG Shelves (CGSh; ±2000 bp flanking a CGS) and (iv) “Open Sea” (OS; outside the CGSh) [34].

DNAm quality-control plots were generated to evaluate read coverage, depth and distribution across sites for each sample. In all samples, a threshold for sufficient single cytosine coverage of a minimum of 10 reads was applied, with a removal of extremely high-coverage sites (>99.9th percentile) [34]. We assessed the 5-methylcytosine: cytosine (5MC:C) ratio at the DMC level with predefined effect size of minimal DNAm difference (DmD, ∆β) ≥ 25% and overdispersion handling (MN method) [34]. Continuous variables were compared using a two-sided Student’s *t*-test or Wilcoxon test, as appropriate according to data distribution. Categorical variables were compared using Fisher’s exact test or the chi-square test, as appropriate. We set a baseline statistical significance (*p*-value) of <0.05. To limit false-positive results, we further used q-value (<0.01) for multiple testing correction based on the false discovery rate (FDR) and Benjamini–Hochberg’s correction (adjusted *p*-value < 0.05; *p*.adjust). Medians with an interquartile range (IQR, q1–q3) and percentage values were used to show quantitative data. We displayed unsupervised principal component analysis (PCA) and heatmaps to illustrate sample clustering along DNAm patterns found to differentiate groups [34]. DMCs and DMRs were depicted on volcano plots and in tables, while GSEs were depicted using lollipop graphs and tables. The databases for GSE included Gene Ontology (GO) and the Kyoto Encyclopedia of Genes and Genomes (KEGG) [34].

### 2.5. Sensitivity Analyses

Additional sensitivity analyses were performed to assess the robustness of the principal findings with respect to selected potential confounding factors, particularly smoking status, age, and longitudinal changes in peripheral leukocyte composition. To minimize the potential influence of smoking, the baseline TED-P1 versus HC comparison was repeated after restriction to never-smokers (7 TED-P1 and 7 HC). Because a substantial age imbalance remained in this restricted subgroup, age was additionally incorporated as a covariate in the differential methylation analysis. The robustness of the observed methylation pattern was assessed using genome-wide differential methylation results and complementary graphical diagnostics. Finally, potential longitudinal changes in peripheral leukocyte composition were evaluated using paired routine complete blood counts available at TED-P1 and TED-P2.

### 2.6. Use of Generative Artificial Intelligence

Generative artificial intelligence (ChatGPT, GPT-5.6, OpenAI) was used during manuscript revision to assist with English-language editing, improve clarity and readability, and support the organization of revised text and figures. All scientific content, analyses, interpretations, and conclusions were independently reviewed and approved by the authors.

## 3. Results

### 3.1. Characteristics of the Subjects

A total of 24 patients with active, moderate-to-severe TED were recruited. All patients had previously received antithyroid drugs for GD, either alone or in combination with definitive treatment (50%; Table 1). Baseline clinical characteristics of TED patients are summarized in Table 1. No significant differences were observed between future responders and non-responders at TED-P1. Following ivGC therapy (TED-P2), a significant decrease in CAS, as well as lid retraction, aperture change and proptosis, was observed in TED responders, who accounted for 58.33% of the cohort (Figure 1 and Table 1).

The healthy subject group (HC) consisted of seven non-smoking individuals without AITD (6 females, 1 male; median age: 29 years) with a median TSH level of 2.5 (2.00–3.00) µIU/mL.

### 3.2. Genome-Wide DNAm in TED Patients at Baseline (TED-P1) and Post-ivGCs (TED-P2)

A total of 55 RRBS libraries, comprising 24 TED-P1, 24 TED-P2 and 7 HC samples, were included in the sequencing analysis. All libraries included in the downstream methylation analysis met the predefined quality-control criteria. Detailed sequencing and quality-control metrics for individual libraries are available from the corresponding author upon reasonable request.

PCA demonstrated separation between TED-P1 and HC samples (Figure 2a). The average DNAm difference between groups was −13.14% and was observed across all autosomal chromosomes (Figure 2b). Promoters and CGIs were the most frequently represented genomic contexts among detected DMCs at both TED-P1 and TED-P2 (Figure 3a–d). At TED-P2, the average DmD was −12.51%, with a modified genome-wide distribution pattern. PCA continued to show separation between TED-P2 and HC, although the clustering pattern differed from baseline and a greater proportion of post-treatment samples overlapped with controls (Figure 2).

### 3.3. Site-Specific DNAm in TED Patients at Baseline (TED-P1) and Post-ivGCs (TED-P2)

Compared with HC, TED-P1 patients presented with predominant hypomethylated patterns on differential heatmap analysis (Figure 4a). Among 14,357 detected DMCs, 2894 reached statistical significance (q < 0.01), of which 99.17% were hypomethylated (Figure 4b). By reference to HGNC nomenclature, 2786 DMRs were identified. In the TED-P2 vs. HC comparison, 3636 CpG sites were detected, including 770 significant DMCs, with hypomethylation remaining the dominant pattern (Figure 4c,d), and 767 DMRs were identified according to HGNC nomenclature. The top 15 DMRs for TED-P1 and TED-P2 are presented in Table 2 and Table 3, respectively, while the remaining CpG sites for these analyses are shown in Tables S1 and S2.

### 3.4. Functional Enrichment Analysis Based on Aberrant DMCs in TED Patients at Baseline (TED-P1) and Post-ivGCs (TED-P2)

Functional enrichment analysis was performed using significant CpGs differentiating TED patients from HC. In the TED-P1 versus HC comparison, 26 significantly enriched GO terms and 16 KEGG pathways were identified (adjusted *p* < 0.05; Figure 5a,b; Tables S3 and S4). Enriched categories included pathways related to visual system development, cell-cycle regulation, Rap1 signaling, and calcium signaling. In the TED-P2 versus HC comparison, eight significant GO terms were identified, including neuronal-related pathways associated with GABAergic differentiation (Table S5).

### 3.5. Sensitivity Analyses

Restriction of the baseline analysis (24 TED-P1 and 7 HC) to never-smokers (7 TED-P1 and 7 HC) did not materially alter the predominant DNAm pattern (Figure 2a–d, Figure 4a–d and Figure S3a–c). The analysis remained characterized by a marked predominance of hypomethylation, with 8251 hypomethylated and 29 hypermethylated DMCs meeting the predefined thresholds. Hypomethylation was consistently observed across all autosomal chromosomes, and PCA continued to demonstrate separation between TED-P1 and HC, although individual variability was evident (Figure S3a–c).

Because restriction of the analysis to never-smokers did not eliminate the age imbalance between TED-P1 patients and HC, age was additionally included as a covariate in the differential methylation model. The predominant hypomethylation pattern remained evident after adjustment for age. The genomic inflation factor for the age-adjusted analysis was λ = 2.745, based on 56,865 CpG sites. To provide an empirical reference for this value, permutation analyses were performed using randomly reassigned group labels. The corresponding λ values range was 0.730–1.410 (median: 0.96). Thus, the λ observed in the age-adjusted analysis exceeded the range obtained in all permutation analyses. Accordingly, the predominant TED-associated methylation pattern persisted after accounting for both smoking status and age.

In addition, paired peripheral blood-cell counts were available for 15 patients at TED-P1 and TED-P2. No significant longitudinal changes were observed in neutrophil, lymphocyte or monocyte proportions, or in NLR (all *p* > 0.05, Wilcoxon signed-rank test; Table S7 and Figure S4a–c).

### 3.6. Genome-Wide and Site-Specific DNAm in TED Patients: Response to the ivGC Treatment and Clinical Associations

PCA did not demonstrate clear separation between responders and non-responders (Figure 6). The comparison between responders and non-responders did not reveal a consistent DNAm signature associated with clinical treatment response in this cohort. In an exploratory analysis, TED-P1 and TED-P2 samples were additionally compared within individual patients, revealing numerous significant within-patient DNAm changes after treatment. Nonetheless, the overall numbers of significant DMCs did not differ between responders and non-responders (Table 4).

There were no significant findings as regards genome-wide and site-specific DNAm associations with prior RAI, thyroid surgery or smoking stratification within the TED-P1 patients. In the exploratory analysis according to cumulative ivGC exposure (7.5 vs. 4.5 g ivMP), 37 significant DMCs were identified, with a predominance of hypermethylation (Supplementary Information; Figure S2, Table S6).

## 4. Discussion

### 4.1. TED Patient Characteristics and Clinical Outcome

The demographic characteristics of our cohort were broadly comparable to those reported in the EUGOGO PREGO III study, although smoking and previous treatment for GD, including ATD, RAI, and thyroidectomy, were more frequent in our cohort [36]. Reported response rates to ivGC therapy vary according to the treatment regimen and response criteria used, ranging from approximately 58–83% for improvement in disease activity and 20–60% for measures of disease severity, including proptosis [8,37]. Using the modified Composite Index, 58.33% of patients in our cohort were classified as responders.

### 4.2. Genome-Wide DNAm Patterns in TED at Baseline (TED-P1) and Post-ivGCs (TED-P2)

Xin et al. applied RRBS to assess genome-wide DNAm in peripheral whole blood from a Chinese cohort comprising 6 TED patients and 6 healthy controls [26]. Consistent with our findings, PCA demonstrated distinct clustering of TED and control samples [26]. In addition, we observed a post-treatment shift in the clustering pattern of TED samples following ivGC (TED-P2 vs. HC).

We also identified predominant genome-wide DNA hypomethylation in TED-P1 patients compared with healthy controls. This finding is also in line with the report by Xin et al. [26]. Although the number of DMCs decreased after ivGC therapy in our patients, DNA hypomethylation remained the dominant pattern. Predominant genome-wide hypomethylation has likewise been reported in GD and may partially normalize following ATD or RAI treatment [38]. Finally, the overall distribution of DMCs across genomic features and CpG contexts remained broadly comparable between TED-P1 and TED-P2 when each was compared with the HC group.

### 4.3. Site-Specific DNAm Alterations in TED at Baseline (TED-P1) and Post-ivGCs (TED-P2)

Recent years have brought a growing body of evidence regarding site-specific DNAm alterations in GD, mostly in immunoregulatory loci, and our results are consistent with these findings [21,26,39,40]. We also found hypomethylated CpGs mapped on AITD-related loci including HLA-DRB5 and BTB and CNC homology 1 (BACH2) [41].

Xin et al. identified a total of 841 DMRs differentiating TED patients and healthy subjects [26]. We identified 98 DMRs that overlapped with those reported by Xin et al., including DRD4, LHX2 and HOXB13 [26]. As expected, some discrepancies in DNAm status were observed in selected replicated DMRs [26]. These differences may be explained by non-identical CpG positions annotated to the same genomic features across studies. Zhang et al. described numerous DMCs in patients with GD, pre-orbitopathy, and TED, mostly in hyperthyroid and predominantly non-smoking cohorts, without inclusion of healthy controls [42]. In our analysis, several overlapping DMRs were identified compared with the study by Zhang et al., including loci related to zinc-finger proteins, long intergenic non-protein coding RNA, and mitogen-activated protein kinases for TED-P1 vs. HC analysis [42]. In contrast to Zhang et al.’s results, most DMRs found in our study were hypomethylated and located in CGIs [42]. These differences may reflect variation in study populations, as well as methodological differences, including the use of microarray-based methylation profiling, flow-sorted lymphocyte subsets, and different methylation thresholds [42]. In our study, the analytical strategy was designed to identify relatively large methylation differences, with a predefined minimum effect-size threshold of DNAm difference ≥ 25%. Consequently, smaller methylation differences were outside the predefined scope of DMC detection rather than simply being missed because of limited statistical power.

The pathogenesis of TED involves complex interactions between immune cells, orbital fibroblasts, and extracellular matrix components, accompanied by signaling pathways mediated by IGF1-R (Insulin-like Growth Factor 1 Receptor) and TSH-R [43]. In the TED-P1 patients, aberrant DNAm was detected in loci previously implicated in immune regulation and tissue remodeling, including forkhead box O3 (FOXO3) and Lysyl Oxidase Like 3 (LOXL3) [44,45]. DNAm alterations were also identified in genes encoding proteins targeted by established or emerging therapies, including IGF1R, the molecular target of teprotumumab, and IL6R, which is relevant to IL-6 receptor blockade with tocilizumab [8]. DNAm alterations involving CD55 may also warrant further investigation in the context of emerging bispecific therapeutic strategies [46].

### 4.4. Gene-Enrichment Findings in TED at Baseline (TED-P1) and Post-ivGC (TED-P2)

GSE analysis revealed multiple biological pathways associated with the detected DMCs, including kinase signaling, calcium-dependent pathways and transforming growth factor-beta responses. Interestingly, categories related to visual system development were also identified in the enrichment analyses. Although potentially relevant in the context of TED, these findings require cautious interpretation. The present analysis was performed in peripheral whole-blood DNA, and enrichment of genes annotated to developmental or visual-system-related pathways does not necessarily indicate a direct functional relationship with orbital tissue or TED pathogenesis. Such genes may have pleiotropic functions across different tissues and biological processes. Therefore, these enrichment findings should primarily be regarded as hypothesis-generating observations requiring functional and tissue-specific validation.

Previous studies reported less consistent enrichment results [26,39,40,47,48]. Xin et al. did not identify significant KEGG pathways, while GO terms were mainly related to cellular and membrane-associated processes [26]. In contrast, our results show similarities to pathways reported in GD by Cai et al., including cell-cycle-related signaling or glioma [39]. Other authors have demonstrated enrichment of immune-related pathways, particularly those associated with T-cell receptor signaling, when DNAm was analyzed in selected lymphocyte subsets [40]. According to Zhang et al., DNAm alterations related to hypoxia signaling and CD4+ T-cell differentiation have also been described in the pre-orbitopathy stage of GD [42].

### 4.5. DNAm Alterations in TED: The ivGC Treatment Response and Clinical Associations

#### 4.5.1. DNAm Patterns and Clinical Response to ivGC Therapy

The longitudinal analytical strategy focused on within-patient DNAm changes at the individual-sample level, complemented by between-group analyses according to clinical treatment response, rather than on a cohort-level paired differential methylation model. In our study, PCA plots did not reveal clear clustering according to response to ivGC, and no consistent DMCs distinguishing responders from non-responders were identified. This may reflect the heterogeneity of treatment response and the limited ability of genome-wide analysis to detect uniform single-site changes across all patients. The absence of consistent cohort-level DMCs in the differential analysis should be interpreted in the context of the stringent single-CpG analytical strategy applied to a small and biologically heterogeneous cohort. Different patients may exhibit treatment-associated methylation changes within the same gene or regulatory region without necessarily showing concordant changes at exactly the same CpG position. The TED-P2 versus HC comparison should therefore be regarded as complementary to, rather than a substitute for, the paired TED-P1 versus TED-P2 analysis. Individual-level analyses nevertheless revealed treatment-associated DNAm changes involving genes related to immune, metabolic, and regulatory processes. However, the overall number of significant DMCs did not differ significantly between responders and non-responders. Treatment response to ivGC is likely multifactorial and may be influenced by clinical heterogeneity, concomitant treatment, and differences in leukocyte composition [38,39].

#### 4.5.2. DNAm Patterns According to Cumulative ivGC Exposure

In an exploratory analysis, patients receiving the higher cumulative ivMP dose showed a predominance of post-treatment hypermethylation. However, only six patients received the 7.5 g regimen, and treatment intensity was related to clinical disease characteristics. Therefore, this observation should not be interpreted as evidence of a dose-dependent epigenetic effect. Rather, it represents a preliminary finding that warrants evaluation in larger cohorts specifically designed to assess the relationship between glucocorticoid exposure and DNAm changes [27,28,29,30].

#### 4.5.3. Clinical Heterogeneity and Treatment-Related Factors

The TED cohort was clinically heterogeneous, including differences in previous treatment exposure. We therefore explored several potential clinical sources of this heterogeneity individually. No significant genome-wide or site-specific DNAm associations were identified with prior RAI treatment or thyroid surgery at baseline, while no significant association with ORTH was identified in the post-treatment analysis. Nevertheless, the absence of significant associations with these individual clinical factors does not exclude residual confounding, particularly given the limited sample size.

#### 4.5.4. Sensitivity Analyses and Potential Confounding Factors

Age, smoking, and peripheral leukocyte composition represent important potential confounders in whole-blood DNAm studies. Importantly, the predominant genome-wide hypomethylation pattern remained evident after restricting the analysis to never-smokers and was also retained in the subsequent age-adjusted analysis of this subgroup.

Cigarette smoking is a well-established environmental risk factor for GD and an even stronger determinant of TED severity, as well as an established modifier of DNA methylation [49,50]. Consequently, complete separation of disease-related and smoking-related epigenetic signals is challenging in real-world TED cohorts. However, the predominance of hypomethylation and separation between TED and HC persisted in the analysis restricted to never-smokers, indicating that the principal disease-associated methylation pattern was not solely attributable to smoking status. Although the genomic inflation factor remained elevated, λ should not be interpreted in isolation as evidence of residual confounding. In the age-adjusted analysis, the observed λ exceeded the entire range obtained from random group-label permutations, while the methylation changes remained strongly directional, with a marked predominance of hypomethylation. Taken together, these observations suggest that the deviation from the null distribution may at least partly reflect a widespread disease-associated methylation signal rather than random statistical inflation alone. In the present study, smoking status was not significantly associated with genome-wide DNAm differences between TED patients at baseline. This might reflect the dynamic and potentially reversible nature of already described, smoking-related DNAm changes, as well as the influence of disease stage, thyroid function and duration of smoking exposure [51,52,53]. A previous study by Zhang et al. also suggested that DNAm alterations may vary during progression from GD to TED and may depend on disease stage [42].

GCs exert both genomic and non-genomic immunomodulatory effects, and previous studies have shown that GC exposure may alter DNAm patterns in peripheral blood cells [27,28,29,30]. Dexamethasone has also been associated with changes in regulatory T-cell function and T-helper-cell composition, further illustrating the potential interaction between glucocorticoid exposure, immune-cell composition, and epigenetic regulation [27]. To explore this potential source of bias, we additionally analyzed routinely available paired peripheral blood counts. Among patients with available paired measurements, we observed no significant systematic changes in total leukocyte count, neutrophil, lymphocyte or monocyte counts and proportions, or NLR between TED-P1 and TED-P2. This observation provides some reassurance that the longitudinal DNAm findings were not accompanied by a major shift in routinely measured leukocyte composition. However, routine blood counts cannot capture changes in specific leukocyte subpopulations and do not substitute for methylation-based cell-type deconvolution. Residual confounding by cellular composition therefore cannot be excluded.

### 4.6. Strengths and Limitations of the Study

To our knowledge, this is among the few studies applying RRBS to characterize genome-wide DNA methylation in thyroid eye disease and the first to evaluate treatment-associated methylation dynamics following standard intravenous glucocorticoid therapy in a prospectively followed cohort. The choice of a DNA methylation profiling platform requires careful consideration of genomic coverage, resolution, scalability, cost, and analytical complexity. DNA methylation microarrays are highly standardized, making them particularly suitable for large cohorts and translational studies; however, they are inherently limited to a predefined and numerically restricted set of CpG loci, in contrast to sequencing-based approaches that generate millions of reads per sample [16,17,18]. Enrichment-based strategies, exemplified by MeDIP-seq, enable broad regional interrogation of the methylome at moderate cost, although they lack single-CpG resolution and may preferentially capture CpG-density-dependent regions [16,17,18]. By comparison, RRBS provides quantitative single-CpG resolution across CpG-enriched genomic regions and enables substantially greater sequencing depth within the represented fraction of the genome at a lower sequencing burden than WGBS. Nevertheless, its reduced representation is inherently non-uniform. The use of restriction digestion at CCGG sites and subsequent fragment size selection preferentially enriches CpG-rich genomic regions, while CpG-poor intergenic and distal regulatory regions are less comprehensively represented. Moreover, the combination of restriction-based library preparation and bisulfite conversion produces a characteristic non-random nucleotide composition [16,17,18].

Several limitations should be acknowledged. First, although DNAm is commonly assessed in whole-blood leukocytes, cell-type-specific differences in methylation cannot be excluded [21,23,24,25,26,40]. Changes in leukocyte composition during ivGC therapy may influence whole-blood DNAm patterns [54]. We partially addressed this issue by analyzing paired routine blood counts where available and found no significant systematic changes in the major routinely measured leukocyte populations between TED-P1 and TED-P2. Nevertheless, blood counts were not available for the entire cohort, and routine hematological measurements cannot substitute for formal methylation-based cell-type deconvolution. Residual cell-composition confounding therefore remains an important limitation of the present study. Second, the clinical heterogeneity of TED should be considered. Variability in disease course, prior GD treatment and the use of ORTH in more severe cases could have affected DNAm profiles, as treatment-related epigenetic changes have been reported previously [30,38,55,56,57]. Another important limitation is the relatively small sample size, particularly the limited number of HC and the smaller subgroups used in the exploratory analyses. These sample sizes limit statistical power, particularly for subgroup analyses, and increase the possibility that smaller or heterogeneous methylation differences remained undetected. Nevertheless, despite its limited size, the HC group showed relatively homogeneous genome-wide DNAm profiles and compact clustering in PCA, in contrast to the greater inter-individual heterogeneity observed among TED patients. The latter is biologically and clinically plausible given the heterogeneous disease course and differences in previous treatment exposure within the TED cohort. Moreover, the predefined DNAm threshold focused the analysis on relatively large methylation differences.

## 5. Conclusions

The present study identified genome-wide and site-specific DNAm alterations in patients with moderate-to-severe TED and differences in DNAm patterns following ivGC therapy. Exploratory findings also suggested variation according to cumulative ivMP exposure; however, the limited size of the higher-dose subgroup precludes conclusions regarding a dose-dependent effect. We did not identify a consistent DNAm signature associated with clinical treatment response in this cohort. These observations warrant validation in larger, independent, and longitudinally characterized cohorts.

The baseline differences in age and smoking status and the use of whole-blood DNA represent potential sources of confounding. Although additional sensitivity analyses addressing smoking status and age, together with paired evaluation of routinely measured leukocyte composition, supported the robustness of the principal findings, residual confounding cannot be excluded. In particular, the small HC group and limited age overlap, as well as the absence of formal methylation-based cell-type deconvolution, should be considered when interpreting the results. Given the limited data on epigenetic mechanisms in TED, further studies are required to confirm these findings and to better define the potential role of DNAm in TED pathogenesis and treatment outcomes.

## Figures and Tables

**Figure 1:**
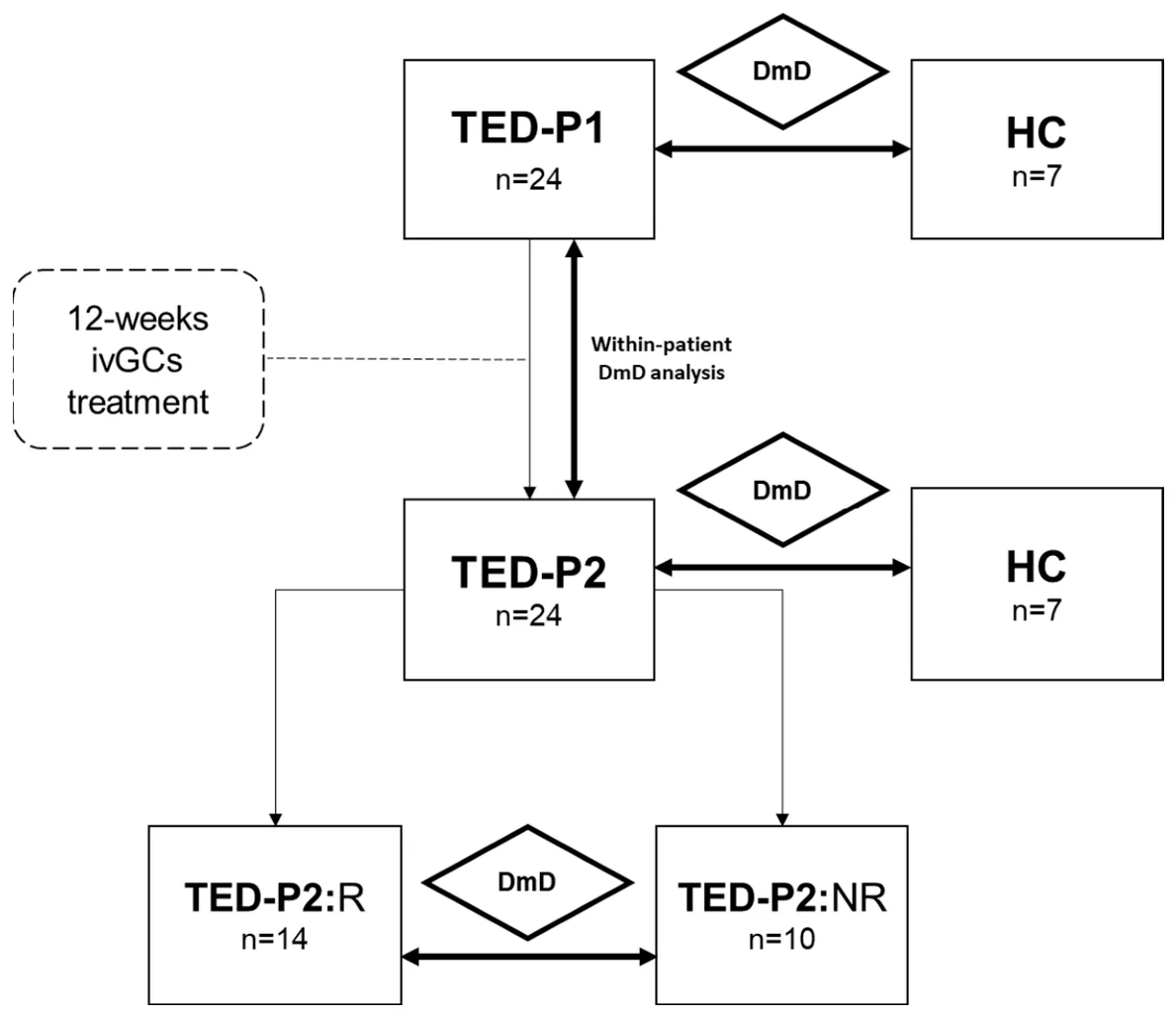
The study flowchart illustrates the protocol timeline for TED patients. Assessment points are marked as TED-P1 and TED-P2, corresponding to evaluations performed either before or after ivGC treatment, respectively, and indicate samples and medical data collection. Abbreviations: DmD—DNAm difference, HC—healthy controls, ivGC—intravenous glucocorticoids, TED-P1—thyroid eye disease patients before ivGC therapy, TED-P2—thyroid eye disease patients after ivGC therapy, TED-P2:NR—thyroid eye disease patients after ivGC therapy with no response to treatment, TED-P2:R—thyroid eye disease patients after ivGC therapy with response to treatment.

**Figure 2:**
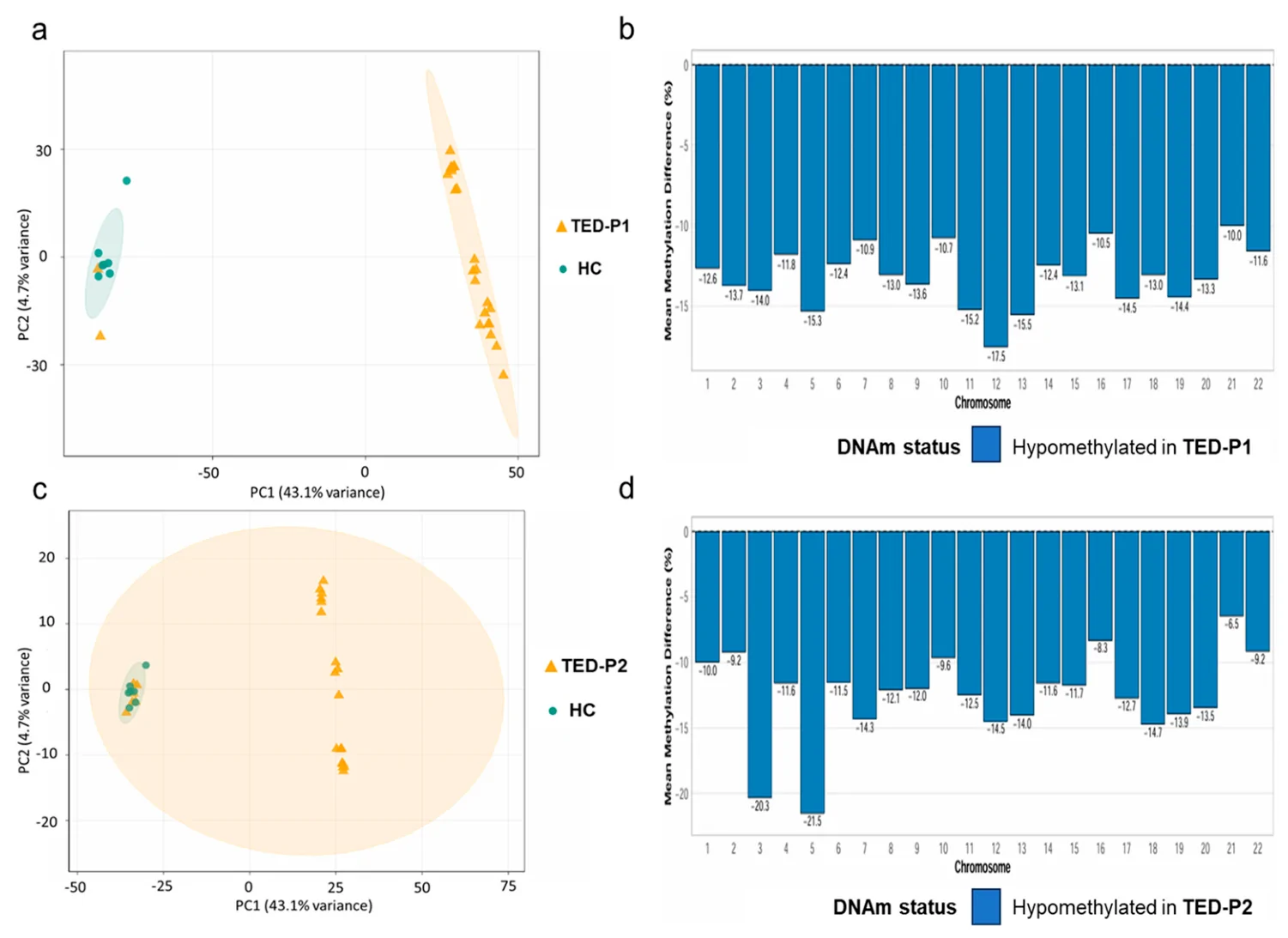
(**a**–**d**). The genome-wide DNAm presentation in either TED-P1 or TED-P2 patients (cases) and HC (controls). (**a**,**c**) Principal component analysis plots showing sample grouping of TED-P1 (**a**) and TED-P2 (**c**) patients (orange triangles) and HC (green dots), based on DNAm levels of individual CpGs. (**b**,**d**) DNAm differences stratified by each autosomal chromosome in TED-P1 (**b**) and TED-P2 (**d**) patients vs. HC. Abbreviations: TED-P1—thyroid eye disease patients before ivGC therapy, TED-P2—thyroid eye disease patients after ivGC therapy, HC—healthy controls, PC1—principal component 1, PC2—principal component 2.

**Figure 3:**
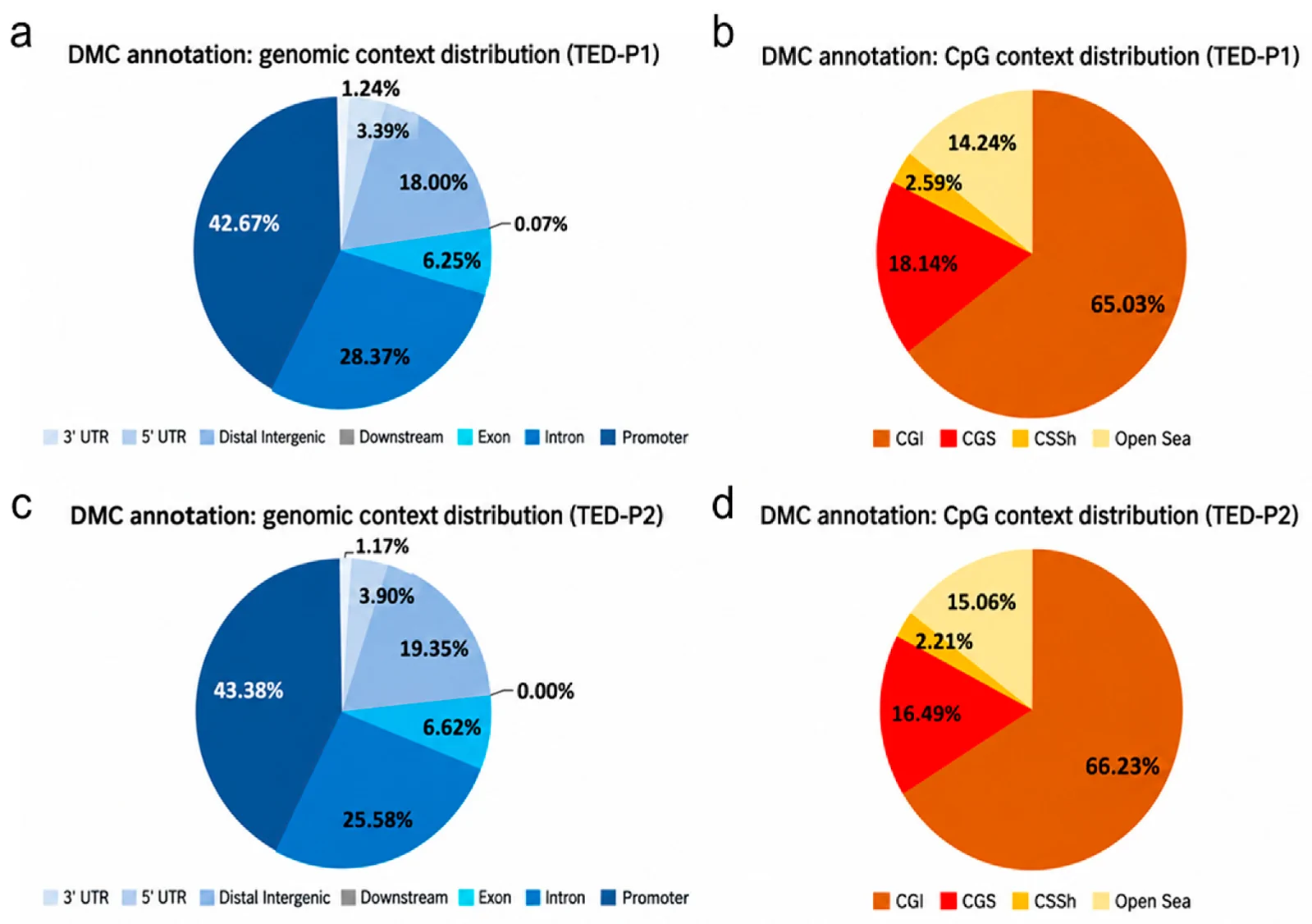
(**a**–**d**). DMCs distribution across genome and CpG context. Graphs illustrate percentage of significant DMCs found in either TED-P1 (**a**,**b**) or TED-P2 (**c**,**d**) groups vs. HC. Abbreviations: 3′UTR—3′ untranslated regions, 5′UTR—5′ untranslated regions, CGI—CpG Islands, CGS—CpG Shores, CGSh—CpG Shelves, CpG—cytosine-phosphate-guanosine dinucleotides, DMCs—differentially methylated cytosines, HC—healthy controls, TED-P1—thyroid eye disease patients before ivGC therapy, TED-P2—thyroid eye disease patients after ivGC therapy.

**Figure 4:**
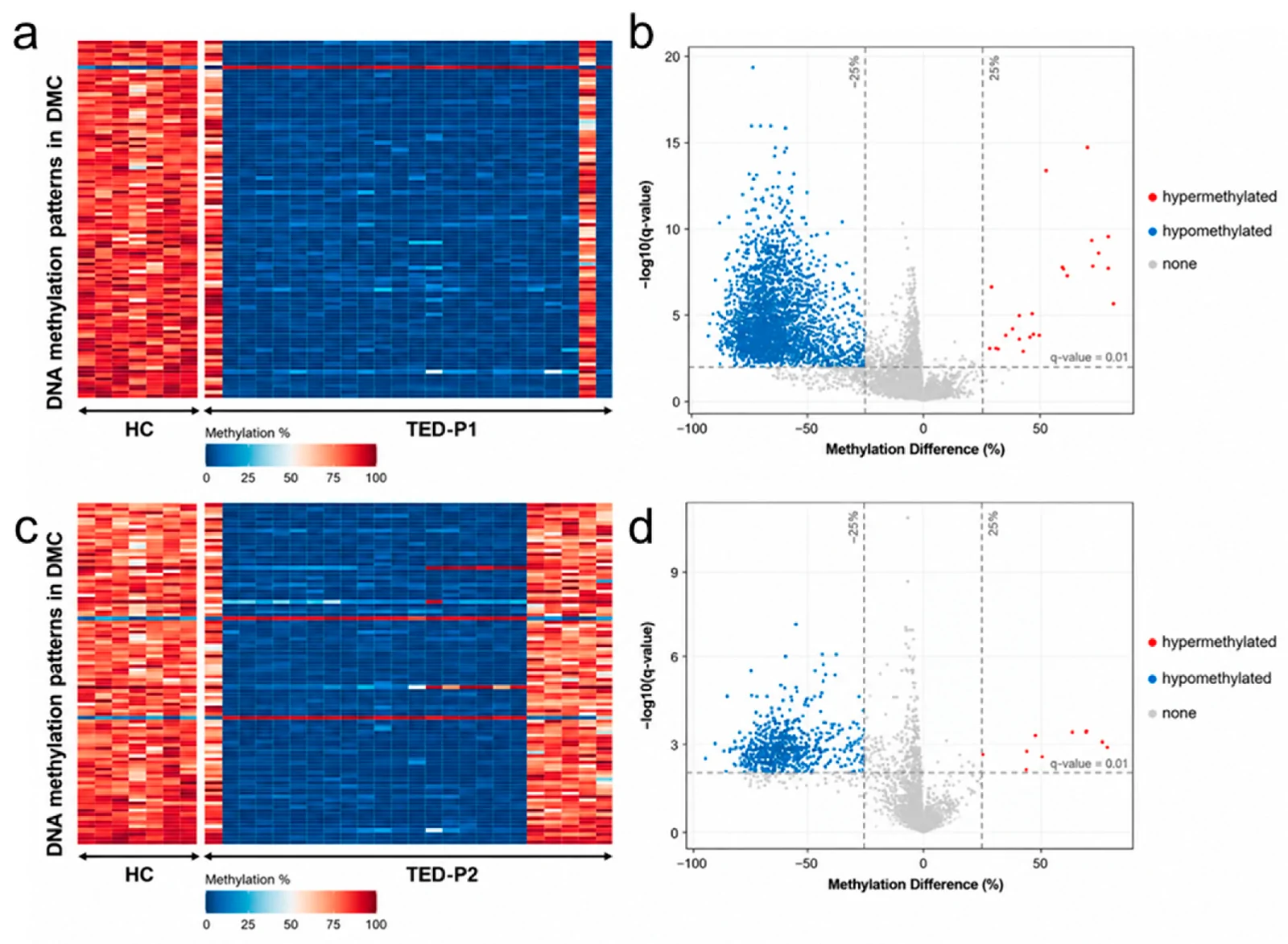
(**a**–**d**). (**a**) Differential DNAm analysis for TED-P1 or TED-P2 patients and HC, respectively. (**a**,**c**) Heatmaps presenting clusters of DNAm patterns (rows, top 100 DMCs) in studied subjects (arrows, columns); hypomethylation and hypermethylation are expressed in blue and red, respectively; color intensity of the pattern indicates its DNAm level in line with a scale below the chart. (**b**,**d**) Significant DMCs of HC vs. TED analysis; q-value 0.01 (a horizontal dashed line). Volcano plot presents differential methylation differences (threshold 25%; vertical dashed lines) on *x*-axis and −log10(q-value) on *y*-axis. Non-grey colored points represent significant hypomethylated (blue; b, *n* = 2870; d, *n* = 760) and hypermethylated (red; b, *n* = 24; d, *n* = 10) CpG sites, respectively. Distance from center indicates effect size magnitude; DMCs of highest rank are located at the top of the figure. Abbreviations: DMC—differentially methylated cytosine, HC—healthy controls, log—logarithm, TED-P1—thyroid eye disease patients before ivGC therapy, TED-P2—thyroid eye disease patients after ivGC therapy.

**Figure 5:**
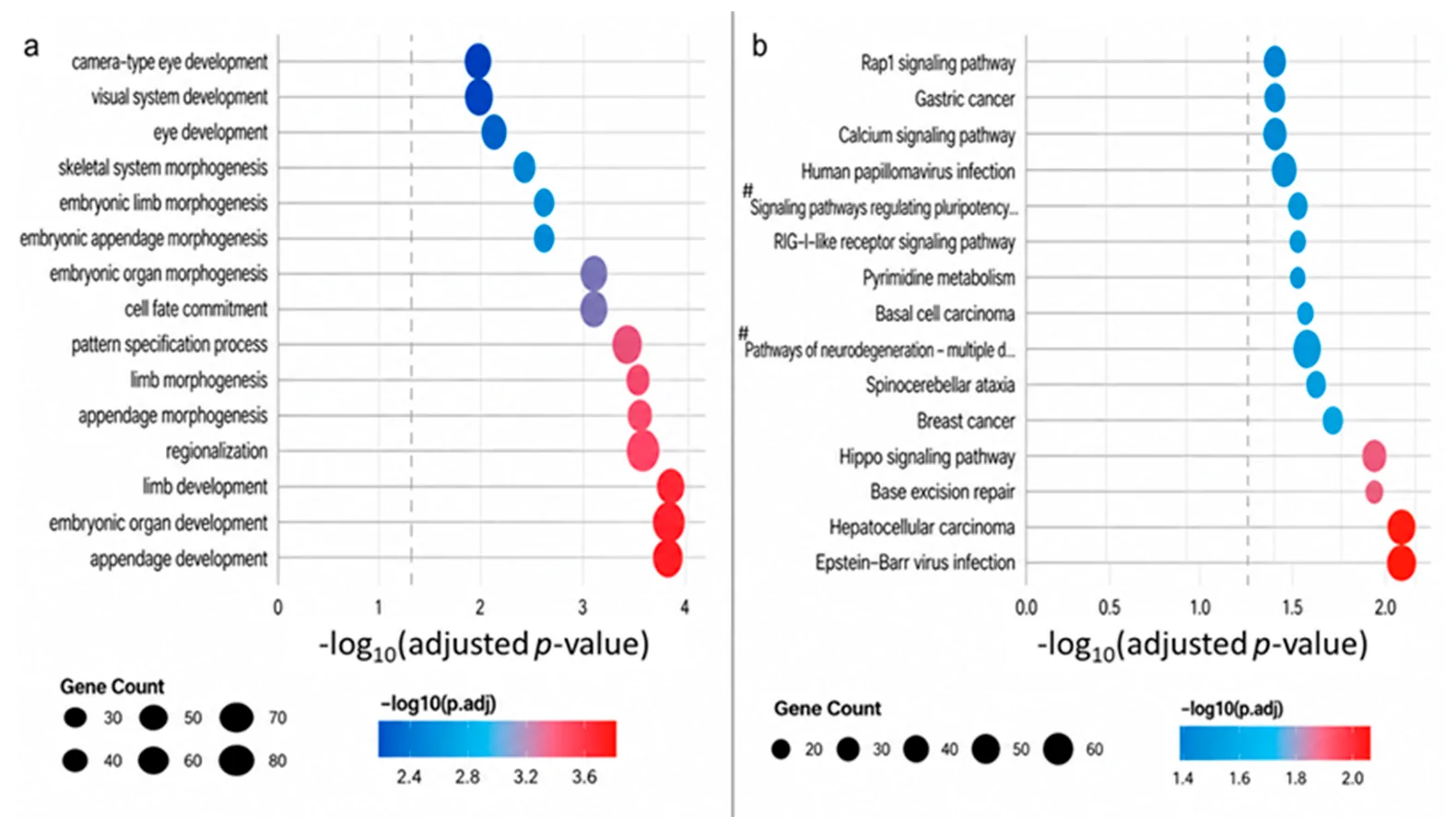
(**a**,**b**). Functional analysis (TED-P1 vs. HC) of the top 15 identified biological processes and pathways referred to GO (**a**) and KEGG (**b**) databases in the form of a lollipop plot. Each graph illustrates significance as −log10(adjusted *p*-value) on *x*-axis and pathways list on *y*-axis. A dashed vertical line represents a statistical significance level, while scaled color saturation was used to reflect significance value; the number of genes involved in a particular metabolic pathway is represented by point diameter and value (Gene Count). Abbreviations: GO—Gene Ontology, KEGG—Kyoto Encyclopedia of Genes and Genomes, log—logarithm. ^#^—Full pathway names: “Pathways of neurodegeneration—multiple diseases” and “Signaling pathways regulating pluripotency of stem cells”.

**Figure 6:**
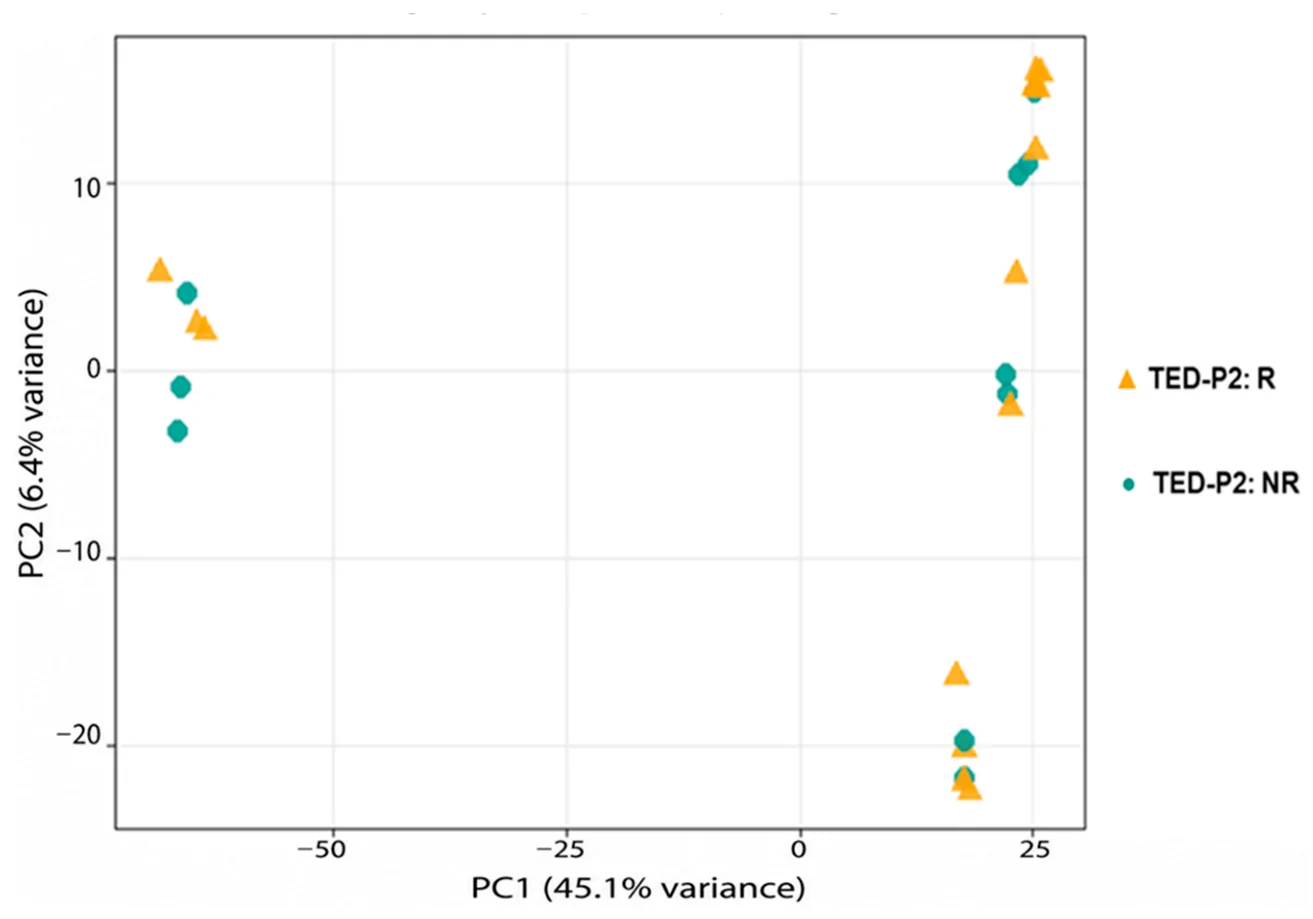
Principal component analysis plot showing TED patient samples (TED-P2) clustering in accordance with ivGC treatment response. Abbreviations: PC1—principal component 1, PC2—principal component 2, TED-P2:NR—thyroid eye disease patients after ivGC therapy with no response to treatment, TED-P2:R—thyroid eye disease patients after ivGC therapy with response to treatment.

**Table 1:** Baseline clinical characteristics of patients with thyroid eye disease.

Variable	TED, (TED-P1, All; *n* = 24)	Responders (*n* = 14)	Non-Responders (*n* = 10)	*p*-Value
**Patient characteristics**				
Age, years	49.5 (41.5–65)	51.5 (43–66)	49.5 (35–64)	0.52
Female sex	19 (79.17)	12 (85.71)	7 (70.0)	0.615
Smoking history	17 (70.83)	10 (71.43)	7 (70.0)	1.00
Family history of AITD	8 (33.33)	5 (38.46)	3 (30.0)	1.00
**Previous Graves’ disease treatment**				
Antithyroid drugs	24 (100)	14 (100)	10 (100)	1.00
Radioactive iodine	5 (20.83)	2 (14.29)	3 (30.0)	0.614
Thyroidectomy	7 (29.17)	2 (14.29)	5 (50.0)	0.085
**Laboratory findings**				
TSH, μIU/mL (TED-P1)	1.35 (0.635–1.99)	1.75 (0.73–2.28)	1.095 (0.40–1.40)	0.107
fT3, pmol/L (TED-P1)	4.365 (3.875–4.56)	4.19 (3.93–4.55)	4.40 (3.82–4.67)	0.790
fT4, pmol/L (TED-P1)	16.1 (14.0–19.63)	14.45 (13.6–17.5)	19.05 (14.7–19.9)	0.120
TRAb, IU/L (TED-P1)	8.165 (3.995–17.05)	6.90 (4.04–14.60)	13.25 (3.95–31.42)	0.334
TRAb, IU/L (TED-P2)	—	2.115 (1.05–3.54)	6.095 (1.95–12.67)	0.057
**TED treatment**				
Orbital radiotherapy	5 (20.83)	3 (21.43)	2 (20.0)	1.00
Total ivGC dose				
7.5 g, *n* (%)	6 (25.0)	5 (35.71)	1 (10.0)	
4.5 g, *n* (%)	18 (75.0)	9 (64.29)	9 (90.0)	0.340
**TED activity**				
CAS (TED-P1)	4 (3–4)	3.5 (3–4)	4 (3–4)	0.878
CAS (TED-P2)	—	1 (0–1)	2 (2–3)	<0.001
**TED severity (TED-P1)**				
Proptosis, mm	22.25 (19–23.5)	19.5 (18–21.5)	23.5 (23–26)	0.003
Lagophthalmos, mm	0 (0–1)	0 (0–1)	0 (0–1)	1.00
Lid retraction, mm	0.25 (0–1)	0 (0–0)	1 (1–2)	<0.001
Lid aperture, mm	10 (9–11)	9.5 (8–11)	11 (11–13)	0.027
Diplopia, *n* (%)	20 (83.33)	13 (92.86)	7 (70.0)	0.272
**TED severity (TED-P2)**				
Proptosis, mm	—	19 (17–20.5)	24.5 (22.5–25.5)	<0.001
Lagophthalmos, mm	—	0 (0–0)	0 (0–1)	0.537
Lid retraction, mm	—	0 (0–0)	1 (0–2)	0.012
Lid aperture, mm	—	9 (7–10)	12 (11–13)	0.007
Diplopia, *n* (%)	—	10 (71.43)	7 (70.0)	1.00

Continuous variables are presented as median (IQR) unless otherwise indicated. Categorical variables are presented as *n* (%). Reference ranges for laboratory parameters were as follows: fT3, 3.1–6.8 pmol/L; fT4, 12–22 pmol/L; TRAb < 1.75 IU/L; TSH, 0.27–4.2 µIU/mL; Abbreviations: AITD—autoimmune thyroid disease, fT3—free triiodothyronine, fT4—free thyroxine, IQR—interquartile range, ivGC—intravenous glucocorticoid, TED—thyroid eye disease, TED-P1—thyroid eye disease patients before ivGC therapy, TED-P2—thyroid eye disease patients after ivGC therapy, TRAb—thyrotropin receptor autoantibodies, TSH—thyroid stimulating hormone. *p*-value < 0.05 (responders vs. non-responders).

**Table 2:** Top 15 annotated differentially methylated regions (DMRs) identified in the comparison between pre-treatment thyroid eye disease (TED-P1) and healthy controls.

Gene/DMR	Chr	Genomic Position	Genomic Annotation	CpG Context	Distance to TSS (bp)	Δβ (%)	*p*-Value	q-Value
*ANP32A*	chr15	69110993	Intron	CGI	2268	−92.76	2.94 × 10^−5^	1.50 × 10^−4^
*RBFOX2*	chr22	36425177	Promoter	CGI	−592	−92.26	3.97 × 10^−6^	2.86 × 10^−5^
*SLCO2A1*	chr3	133749008	Promoter	CGS	−88	−90.54	8.95 × 10^−7^	8.19 × 10^−6^
*HOXD3*	chr2	177027980	Promoter	CGI	−825	−89.57	3.70 × 10^−9^	8.76 × 10^−8^
*U2AF2*	chr19	56165978	5′ UTR	CGI	562	−88.96	4.41 × 10^−6^	3.11 × 10^−5^
*DGCR6*	chr22	18893614	Promoter	CGI	73	−88.89	1.40 × 10^−4^	5.54 × 10^−4^
*RAET1K*	chr6	150326493	Promoter	CGI	−213	−88.26	6.67 × 10^−7^	6.38 × 10^−6^
*FRG1JP*	chr9	68413573	Exon	CGS	40,802	−88.02	2.51 × 10^−13^	4.26 × 10^−11^
*CAB39*	chr2	231578655	5′ UTR	CGS	1098	−87.94	2.22 × 10^−4^	8.25 × 10^−4^
*NDRG1*	chr8	134309256	Promoter	CGI	291	−87.93	1.07 × 10^−6^	9.54 × 10^−6^
*CHAF1B*	chr21	37757378	Promoter	CGI	49	−87.70	2.25 × 10^−3^	6.30 × 10^−3^
*ELAC1*	chr18	48556561	Intron	CGI	62,174	−87.36	6.73 × 10^−5^	2.97 × 10^−4^
*WASHC4*	chr12	105501601	Promoter	CGI	109	−87.32	8.01 × 10^−6^	5.06 × 10^−5^
*NOL4L-DT*	chr20	31215628	Distal intergenic region	OS	40,347	−87.18	1.27 × 10^−6^	1.11 × 10^−5^
*SPRY4*	chr5	141704465	Promoter	CGI	155	−87.10	1.47 × 10^−4^	5.79 × 10^−4^

Δβ represents the difference in DNA methylation between the compared groups and is expressed as percentage points. Negative values indicate lower methylation in the TED group relative to healthy controls. *p*-value < 0.05; q-value (FDR correction) < 0.01. Abbreviations: 5′UTR—5′ untranslated regions, ANP32A—acidic nuclear phosphoprotein 32 family member A, CAB39—calcium binding protein 39, CGI—CpG island, CGS—CpG shore, CHAF1B—chromatin assembly factor 1 subunit B, Chr—chromosome, CpG—cytosine phosphorus guanine dinucleotide, DGCR6—DiGeorge syndrome critical region gene 6, DMR—differentially methylated region, ELAC1—elaC ribonuclease Z 1, FRG1JP—FSHD region gene 1 family member J (pseudogene), HOXD3—homeobox D3, NDRG1—N-myc downstream regulated 1, NOL4L-DT—NOL4L divergent transcript, OS—Open sea, RAET1K—retinoic acid early transcript 1K (pseudogene), RBFOX2—RNA binding fox-1 homolog 2, SLCO2A1—solute carrier organic anion transporter family member 2A1, SPRY4—sprouty RTK signaling antagonist 4, TED-P1—thyroid eye disease patients before ivGCS, TSS—transcription start site, U2AF2—U2 small nuclear RNA auxiliary factor 2, WASHC4—WASH complex subunit 4.

**Table 3:** Top 15 annotated differentially methylated regions (DMRs) identified in the comparison between post-treatment thyroid eye disease (TED-P2) and healthy controls.

Gene/DMR	Chr	Genomic Position	Genomic Annotation	CpG Context	Distance to TSS (bp)	Δβ (%)	*p*-Value	q-Value
*CTBP2*	chr10	126849294	Promoter	CGI	330	−94.13	1.09 × 10^−3^	3.37 × 10^−3^
*SLCO2A1*	chr3	133748744	Promoter	CGI	176	−90.01	1.47 × 10^−4^	1.00 × 10^−3^
*NDRG1*	chr8	134309256	Promoter	CGI	291	−86.22	4.23 × 10^−4^	1.08 × 10^−3^
*PLEKHO1*	chr1	150122339	5′ UTR	CGI	572	−85.52	3.91 × 10^−3^	8.97 × 10^−3^
*FRG1JP*	chr9	68413573	Exon	CGS	40,802	−84.42	5.82 × 10^−7^	2.51 × 10^−5^
*ELAC1*	chr18	48556561	Intron	CGI	62,174	−83.63	3.25 × 10^−4^	1.57 × 10^−3^
*IFT140*	chr16	1660419	Intron	CGI	1690	−83.58	4.79 × 10^−4^	1.95 × 10^−3^
*BARHL2*	chr1	91184191	Promoter	CGI	−1397	−82.35	3.32 × 10^−4^	1.58 × 10^−3^
*VWCE*	chr11	61062576	Promoter	CGI	212	−81.33	1.87 × 10^−3^	5.02 × 10^−3^
*FGFRL1*	chr4	1003077	Distal intergenic region	CGS	−1863	−81.23	3.05 × 10^−4^	1.53 × 10^−3^
*PPP6R1*	chr19	55770611	Promoter	CGI	−573	−81.12	1.11 × 10^−4^	8.43 × 10^−4^
*FOXI2*	chr10	129536065	Intron	CGI	527	−80.99	2.08 × 10^−4^	1.25 × 10^−3^
*TTC8*	chr14	89506853	Distal intergenic region	OS	216,356	−80.73	2.13 × 10^−3^	5.53 × 10^−3^
*PRSS30P*	chr16	2892798	Promoter	CGI	−87	−80.01	2.70 × 10^−4^	1.46 × 10^−3^
*LSP1P5*	chr1	148901956	Intron	CGI	−26,330	−79.09	3.40 × 10^−5^	4.18 × 10^−4^

DmD (Δβ) represents the difference in DNA methylation between the compared groups and is expressed as percentage points. Negative values indicate lower methylation in the TED group relative to healthy controls. *p*-value < 0.05, q-value (FDR correction) < 0.01. Abbreviations: 5′UTR—5′ untranslated regions, BARHL2—BarH like homeobox 2, CGI—CpG island, CGS—CpG shore, Chr—chromosome, CpG—cytosine phosphorus guanine dinucleotide, CTBP2—C-terminal binding protein 2, DMR—differentially methylated region, ELAC1—elaC ribonuclease Z 1, FGFRL1—fibroblast growth factor receptor like 1, FOXI2—forkhead box I2, FRG1JP—FSHD region gene 1 family member J (pseudogene), IFT140—intraflagellar transport 140, LSP1P5—LSP1 pseudogene 5, NDRG1—N-myc downstream regulated 1, OS—Open sea, PLEKHO1—pleckstrin homology domain containing O1, PPP6R1—protein phosphatase 6 regulatory subunit 1, PRSS30P—serine protease 30 (pseudogene), SLCO2A1—solute carrier organic anion transporter family member 2A1, TED-P2—thyroid eye disease patients after ivGCS, TSS—transcription start site, TTC8—tetratricopeptide repeat domain 8, VWCE—von Willebrand factor C and EGF domains.

**Table 4:** Distribution of differentially methylated cytosines stratified by treatment response in individual patients with thyroid eye disease after intravenous glucocorticoid therapy.

Feature	TED-P2, All (*n* = 24)	Responders (*n* = 14)	Non-Responders (*n* = 10)	*p*-Value
**Differentially methylated cytosines**				
Total DMCs	4960 (1069.5–40,323.5)	3971 (1248–39,681)	10,935.5 (891–40,966)	0.838
Hypermethylated DMCs	2215.5 (565–23,493.5)	1744 (640–22,133)	4511.5 (490–25,102)	0.792
Hypomethylated DMCs	2121 (504.5–7937)	2227 (608–8583)	2121 (401–7291)	0.977
**Genomic distribution**				
Promoters	402.5 (86.5–3043)	289 (84–2069)	783.5 (89–4804)	0.747
Introns	1984 (407.5–15,339)	1697.5 (506–16,855)	4497 (309–13,823)	0.838
Exons	515 (114–3409.5)	425.5 (133–3985)	1064 (95–2834)	0.747
Distal intergenic regions	1862 (430–12,000)	1389.5 (484–12,966)	4142.5 (376–11,034)	0.838
Downstream regions	8 (1–50)	5.5 (1–62)	21.5 (1–43)	0.814
3′ UTR	125.5 (20.5–781.5)	106 (24–891)	278.5 (17–672)	0.792
5′ UTR	63 (13.5–564)	54.5 (15–495)	120 (12–761)	0.792
**CpG context**				
CpG islands (CGIs)	944 (177–6707)	687 (203–4312)	1710 (151–9949)	0.792
CpG shores (CGSs)	864.5 (203–7216)	679.5 (237–7164)	1914 (169–7268)	0.792
CpG shelves (CGShs)	332 (67.5–1761.5)	284 (84–1830)	761.5 (51–1693)	0.815
Open sea (OS)	2819.5 (622–14,029)	2306.5 (724–13,331)	6550 (520–14,727)	0.747

Continuous variables are presented as median (IQR). Abbreviations: 3′UTR—3′ untranslated regions, 5′UTR—5′ untranslated regions, CGI—CpG island, CGI—CpG Islands, CGS—CpG Shores, CGSh—CpG Shelves, CpG—cytosine phosphorus guanine dinucleotide, DMC—differentially methylated cytosine, IQR—interquartile range, *p*—*p*-value, TED-P2—thyroid eye disease post ivGC group.

## Data Availability

The datasets are not publicly available because they contain sensitive human participant data. De-identified data may be made available by the corresponding author upon reasonable request. The bioinformatics pipeline used in this study is publicly available in the MethApp GitHub repository: https://github.com/pgasperowicz/MethApp (accessed on 21 September 2026).

## Supplementary Materials

The following supporting information can be downloaded at: https://www.mdpi.com/article/10.3390/genes17101181/s1, Figure S1: A summary of the laboratory procedures applied in the study; Figure S2: (a–d) Exploratory analysis according to cumulative intravenous methylprednisolone dose in TED patients; Figure S3: (a–c) Sensitivity analysis of DNA methylation patterns restricted to never-smokers; Figure S4: (a–d) Paired analysis of peripheral blood-cell composition in TED patients before and after intravenous glucocorticoid therapy; Table S1: All annotated DMRs (HGNC) identified from significant DMCs in the TED-P1 patients and HC comparison; *p*-value < 0.05, q-value < 0.01 (FDR); Table S2: All annotated DMRs (HGNC) identified from significant DMCs in the TED-P2 patients and HC comparison; *p*-value < 0.05, q-value < 0.01 (FDR); Table S3: All identified biological processes and pathways being referred to KEGG database: TED-P1 vs. HC analysis; *p*-value < 0.05, *p*.adjust < 0.05 (multiple comparison correction); Table S4: All identified biological processes and pathways being referred to GO database: TED-P1 vs. HC analysis; *p*-value < 0.05, *p*.adjust < 0.05 (multiple comparison correction); Table S5: All identified biological processes and pathways being referred to GO database: TED-P2 vs. HC analysis; *p*-value < 0.05, *p*.adjust < 0.05 (multiple comparison correction); Table S6: All annotated DMRs (HGNC) identified from significant DMCs in the TED 7.5 g ivGC and TED 4.5 g ivGC patients comparison; *p*-value < 0.05, q-value < 0.01 (FDR); Table S7: Paired analysis of peripheral blood-cell parameters before and after intravenous glucocorticoid therapy in patients with thyroid eye disease.

## Abbreviations

The following abbreviations are used in this manuscript:

AITD	Autoimmune thyroid disease
ATD	Antithyroid drug
BAM	Binary Alignment Map
CAS	Clinical Activity Score
CGI	CpG island
CGS	CpG shore
CGSh	CpG shelf
CpG	Cytosine–phosphate–guanine dinucleotide
DMC	Differentially methylated cytosine
DmD	DNA methylation difference
DMR	Differentially methylated region
DNAm	DNA methylation
DNMT	DNA methyltransferase
EDTA	Ethylenediaminetetraacetic acid
EUGOGO	European Group on Graves’ Orbitopathy
FASTQ	FASTQ sequence file format
FDR	False discovery rate
fT3	Free triiodothyronine
fT4	Free thyroxine
GC	Glucocorticoid
GD	Graves’ disease
GO	Gene Ontology
GSE	Gene Set Enrichment
HC	Healthy control
HGNC	HUGO Gene Nomenclature Committee
ivGC	Intravenous glucocorticoid
ivMP	Intravenous methylprednisolone
KEGG	Kyoto Encyclopedia of Genes and Genomes
ORTH	Orbital radiotherapy
OS	Open Sea
PCA	Principal Component Analysis
PCR	Polymerase chain reaction
q-value	False discovery rate-adjusted *p*-value
RAI	Radioactive iodine
RRBS	Reduced Representation Bisulfite Sequencing
TED	Thyroid eye disease
TED-P1	thyroid eye disease patients before ivGC therapy (Baseline assessment before intravenous glucocorticoid therapy)
TED-P2	thyroid eye disease patients after ivGC therapy (Assessment 12 weeks after completion of intravenous glucocorticoid therapy)
TRAb	Thyrotropin receptor autoantibody
TSH	Thyroid-stimulating hormone
TSHR	Thyroid-stimulating hormone receptor
UTR	Untranslated region

## References

[1] Antonelli A., Fallahi P., Elia G., Ragusa F., Paparo S.R., Ruffilli I., Patrizio A., Gonnella D., Giusti C., Virili C. (2020). Graves’ disease: Clinical manifestations, immune pathogenesis (cytokines and chemokines) and therapy. Best Pract. Res. Clin. Endocrinol. Metab..

[2] Radziszewski M., Kus A., Bednarczuk T. (2023). Genotype-phenotype correlations in Graves’ disease. Best Pract. Res. Clin. Endocrinol. Metab..

[3] Hansen M., Cheever A., Weber K.S., O’Neill K.L. (2023). Characterizing the Interplay of Lymphocytes in Graves’ Disease. Int. J. Mol. Sci..

[4] Lee S.Y., Pearce E.N. (2023). Hyperthyroidism: A Review. JAMA.

[5] Ahn H.Y., Lee J., Lee J.K. (2022). Predictive Model for Graves’ Ophthalmopathy in Patients with New-Onset Graves’ Disease. Thyroid.

[6] Wiersinga W.M., Eckstein A.K., Zarkovic M. (2025). Thyroid eye disease (Graves’ orbitopathy): Clinical presentation, epidemiology, path-ogenesis, and management. Lancet Diabetes Endocrinol..

[7] Bartalena L., Smith T.J. (2025). Treatment of Hyperthyroidism in Graves’ Disease Complicated by Thyroid Eye Disease. J. Clin. Endocrinol. Metab..

[8] Bartalena L., Kahaly G.J., Baldeschi L., Dayan C.M., Eckstein A., Marcocci C., Marinò M., Vaidya B., Wiersinga W.M., EUGOGO (2021). The 2021 European Group on Graves’ orbitopathy (EUGOGO) clinical practice guidelines for the medical management of Graves’ orbitopathy. Eur. J. Endocrinol..

[9] Virdee S., Moledina M., Lee V. (2025). “Real-Life” Utility of the Graves’ Ophthalmopathy-Quality of Life in a Multidisciplinary Thyroid Eye Disease Service. Ophthalmic Plast. Reconstr. Surg..

[10] Kahaly G.J. (2020). Management of Graves Thyroidal and Extrathyroidal Disease: An Update. J. Clin. Endocrinol. Metab..

[11] Razmara E., Salehi M., Aslani S., Bitaraf A., Yousefi H., Colon J.R., Mahmoudi M. (2021). Graves’ disease: Introducing new genetic and epigenetic contributors. J. Mol. Endocrinol..

[12] Manso J., Censi S., Clausi C., Piva I., Zhu Y.H., Mondin A., Pedron M.C., Barollo S., Bertazza L., Midena G. (2023). Circulating miR-146a predicts glucocorticoid response in thyroid eye disease. Eur. Thyroid J..

[13] Mattei A.L., Bailly N., Meissner A. (2022). DNA methylation: A historical perspective. Trends Genet..

[14] Manna S., Mishra J., Baral T., Kirtana R., Nandi P., Roy A., Chakraborty S., Niharika, Patra S.K. (2023). Epigenetic signaling and crosstalk in regulation of gene expression and disease progression. Epigenomics.

[15] Smith Z.D., Hetzel S., Meissner A. (2025). DNA methylation in mammalian development and disease. Nat. Rev. Genet..

[16] Beck D., Ben Maamar M., Skinner M.K. (2022). Genome-wide CpG density and DNA methylation analysis method (MeDIP, RRBS, and WGBS) comparisons. Epigenetics.

[17] Pidsley R., Zotenko E., Peters T.J., Lawrence M.G., Risbridger G.P., Molloy P., Van Djik S., Muhlhausler B., Stirzaker C., Clark S.J. (2016). Critical evaluation of the Illumina MethylationEPIC BeadChip microarray for whole-genome DNA methylation profiling. Genome Biol..

[18] Neefs I., Ibrahim J., Peeters M., Van Camp G., Op de Beeck K. (2025). The technology landscape for detection of DNA methylation in cancer liquid biopsies. Epigenetics.

[19] Li J., Li L., Wang Y., Huang G., Li X., Xie Z., Zhou Z. (2021). Insights Into the Role of DNA Methylation in Immune Cell Development and Autoimmune Disease. Front. Cell Dev. Biol..

[20] Lafontaine N., Shore C.J., Campbell P.J., Mullin B.H., Brown S.J., Panicker V., Dudbridge F., Brix T.H., Hegedüs L., Wilson S.G. (2024). Epigenome-wide Association Study Shows Dif-ferential DNA Methylation of MDC1, KLF9, and CUTA in Autoimmune Thyroid Disease. J. Clin. Endocrinol. Metab..

[21] Cai T., Qin Q., Song R., Zhao J., Wang G., Zhang J. (2021). Identifying and Validating Differentially Methylated Regions in Newly Diag-nosed Patients with Graves’ Disease. DNA Cell Biol..

[22] Rotondo Dottore G., Lanzolla G., Comi S., Menconi F., Mencacci L.C., Dallan I., Marcocci C., Marinò M. (2023). Insights Into the Role of DNA Methylation and Gene Expression in Graves Orbitopathy. J. Clin. Endocrinol. Metab..

[23] Kinoshita R., Inoue N., Iwatani Y., Noguchi Y., Hidaka Y., Watanabe M. (2024). Methylation levels of the IL10 gene in peripheral blood are related to the intractability of Graves’ disease. Clin. Immunol..

[24] Hashimoto H., Watanabe M., Inoue N., Hirai N., Haga E., Kinoshita R., Hidaka Y., Iwatani Y. (2019). Association of IFNG gene methylation in peripheral blood cells with the development and prognosis of autoimmune thyroid diseases. Cytokine.

[25] Hirai N., Watanabe M., Inoue N., Kinoshita R., Ohtani H., Hidaka Y., Iwatani Y. (2019). Association of IL6 gene methylation in peripheral blood cells with the development and prognosis of autoimmune thyroid diseases. Autoimmunity.

[26] Xin Z., Hua L., Shi T.T., Tuo X., Yang F.Y., Li Y., Cao X., Yang J.K. (2018). A genome-wide DNA methylation analysis in peripheral blood from patients identifies risk loci associated with Graves’ orbitopathy. J. Endocrinol. Investig..

[27] Hu Y., Tian W., Zhang L.L., Liu H., Yin G.P., He B.S., Mao X.M. (2012). Function of regulatory T-cells improved by dexamethasone in Graves’ disease. Eur. J. Endocrinol..

[28] Langericht J., Kramer I., Kahaly G.J. (2020). Glucocorticoids in Graves’ orbitopathy: Mechanisms of action and clinical application. Ther. Adv. Endocrinol. Metab..

[29] Wan E.S., Qiu W., Baccarelli A., Carey V.J., Bacherman H., Rennard S.I., Agusti A., Anderson W.H., Lomas D.A., DeMeo D.L. (2012). Systemic steroid exposure is associated with differential methylation in chronic obstructive pulmonary disease. Am. J. Respir. Crit. Care Med..

[30] Jia W.Y., Zhang J.J. (2022). Effects of glucocorticoids on leukocytes: Genomic and non-genomic mechanisms. World J. Clin. Cases.

[31] Kahaly G.J., Bartalena L., Hegedus L., Leenhardt L., Poppe K., Pearce S.H. (2018). 2018 European Thyroid Association Guideline for the Management of Graves’ Hyperthyroidism. Eur. Thyroid J..

[32] Gu H., Smith Z.D., Bock C., Boyle P., Gnirke A., Meissner A. (2011). Preparation of reduced representation bisulfite sequencing libraries for genome-scale DNA methylation profiling. Nat. Protoc..

[33] Bartalena L., Veronesi G., Krassas G.E., Wiersinga W.M., Marcocci C., Marino M., Nardi M., Daumerie C., Baldeschi L., Boboridis K. (2017). Does early response to intravenous gluco-corticoids predict the final outcome in patients with moderate-to-severe and active Graves’ orbitopathy?. J. Endocrinol. Investig..

[34] Gasperowicz P. (2026). MethApp.

[35] Baek I.C., Sim S.Y., Suh B.K., Kim T.G., Cho W.K. (2025). Assessment of XCI skewing and demonstration of XCI escape region based on single-cell RNA sequencing: Comparison between female Graves’ disease and control. BMC Mol. Cell Biol..

[36] Schuh A., Ayvaz G., Baldeschi L., Baretić M., Bechtold D., Boschi A., Brix T.H., Burlacu M.-C., Ćirić J., Covelli D. (2024). Presentation of Graves’ orbitopathy within European Group On Graves’ Orbitopathy (EUGOGO) centres from 2012 to 2019 (PREGO III). Br. J. Ophthalmol..

[37] Burch H.B., Perros P., Bednarczuk T., Cooper D.S., Dolman P.J., Leung A.M., Mombaerts I., Salvi M., Stan M.N. (2022). Management of Thyroid Eye Disease: A Consensus Statement by the American Thyroid Association and the European Thyroid Association. Thyroid.

[38] Guo Q., Wu D., Yu H., Bao J., Peng S., Shan Z., Guan H., Teng W. (2018). Alterations of Global DNA Methylation and DNA Methyltransferase Expression in T and B Lymphocytes from Patients with Newly Diagnosed Autoimmune Thyroid Diseases After Treatment: A Follow-Up Study. Thyroid.

[39] Cai T.T., Muhali F.S., Song R.H., Qin Q., Wang X., Shi L.F., Jiang W.J., Xiao L., Li D.F., Zhang J.A. (2015). Genome-wide DNA methylation analysis in Graves’ disease. Genomics.

[40] Limbach M., Saare M., Tserel L., Kisand K., Eglit T., Sauer S., Axelsson T., Syvänen A.-C., Metspalu A., Milani L. (2016). Epigenetic profiling in CD4+ and CD8+ T cells from Graves’ disease patients reveals changes in genes associated with T cell receptor signaling. J. Autoimmun..

[41] Simmonds M.J. (2013). GWAS in autoimmune thyroid disease: Redefining our understanding of pathogenesis. Nat. Rev. Endocrinol..

[42] Zhang M., Qi X., Zhou X., Liu Y., He M., Wang J., Wang L., Chen Z., Li S., Chen Y. (2025). A Preclinical State of Graves’ Ophthalmopathy Characterized by Hypoxia of T-cells Identified via Multiomics Analysis. J. Clin. Endocrinol. Metab..

[43] Cui X., Wang F., Liu C. (2023). A review of TSHR- and IGF-1R-related pathogenesis and treatment of Graves’ orbitopathy. Front. Immunol..

[44] Zhang L., Ji Q.H., Ruge F., Lane C., Morris D., Tee A.R., Dayan C.M., Ludgate M. (2016). Reversal of Pathological Features of Graves’ Orbitopathy by Activation of Forkhead Transcription Factors, FOXOs. J. Clin. Endocrinol. Metab..

[45] Park S.H., Choi S.H., Park H.Y., Ko J., Yoon J.S. (2024). Role of Lysyl Oxidase-Like Protein 3 in the Pathogenesis of Graves’ Orbitopathy in Orbital Fibroblasts. Investig. Ophthalmol. Vis. Sci..

[46] Lee S.M., Min S.W., Kwon H.S., Bae G.D., Jung J.H., Park H.I., Lee S.H., Lim C.S., Ko B.J., Lee J.C. (2023). Effective clearance of rituximab-resistant tumor cells by breaking the mirrorsymmetry of immunoglobulin G and simultaneous binding to CD55 and CD20. Sci. Rep..

[47] Xin Z., Hua L., Yang Y.L., Shi T.T., Liu W., Tuo X., Li Y., Cao X., Yang F.Y. (2019). A Pathway Analysis Based on Genome-Wide DNA Methylation of Chinese Patients with Graves’ Orbitopathy. BioMed Res. Int..

[48] Shi T.T., Hua L., Xin Z., Li Y., Liu W., Yang Y.L. (2019). Identifying and Validating Genes with DNA Methylation Data in the Context of Biological Network for Chinese Patients with Graves’ Orbitopathy. Int. J. Endocrinol..

[49] Hoang T.D., Stocker D.J., Chou E.L., Burch H.B. (2022). 2022 Update on Clinical Management of Graves Disease and Thyroid Eye Disease. Endocrinol. Metab. Clin. N. Am..

[50] Antonelli A., Ferrari S.M., Ragusa F., Elia G., Paparo S.R., Ruffilli I., Patrizio A., Giusti C., Gonnella D., Cristaudo A. (2020). Graves’ disease: Epidemiology, genetic and environmental risk factors and viruses. Best Pract. Res. Clin. Endocrinol. Metab..

[51] Joehanes R., Just A.C., Marioni R.E., Pilling L.C., Reynolds L.M., Mandaviya P.R., Guan W., Xu T., Elks C.E., Aslibekyan S. (2016). Epigenetic Signatures of Cigarette Smoking. Circ. Cardiovasc. Genet..

[52] Guida F., Sandanger T.M., Castagne R., Campanella G., Polidoro S., Palli D., Krogh V., Tumino R., Sacerdote C., Panico S. (2015). Dynamics of smoking-induced genome-wide methylation changes with time since smoking cessation. Hum. Mol. Genet..

[53] McCartney D.L., Stevenson A.J., Hillary R.F., Walker R.M., Bermingham M.L., Morris S.W., Clarke T.K., Campbell A., Murray A.D., Whalley H.C. (2018). Epigenetic signatures of starting and stopping smoking. EBioMedicine.

[54] Ronchetti S., Ricci E., Migliorati G., Gentili M., Riccardi C. (2018). How Glucocorticoids Affect the Neutrophil Life. Int. J. Mol. Sci..

[55] Sadahiro R., Knight B., James F., Hannon E., Charity J., Daniels I.R., Burrage J., Knox O., Crawford B., Smart N.J. (2020). Major surgery induces acute changes in measured DNA methylation associated with immune response pathways. Sci. Rep..

[56] Muller L., Hoffmann A., Bernhart S.H., Ghosh A., Zhong J., Hagemann T., Sun W., Dong H., Noé F., Wolfrum C. (2024). Blood methylation pattern reflects epigenetic re-modelling in adipose tissue after bariatric surgery. EBioMedicine.

[57] Ma L., Zhang Y., Xu J., Yu Y., Zhou P., Liu X., Guan H. (2025). Effects of Ionizing Radiation on DNA Methylation Patterns and Their Potential as Biomarkers. Int. J. Mol. Sci..

