# Supplementary material for: DNA Methylation Alterations in Patients with Moderate-to-Severe Thyroid Eye Disease Treated with Intravenous Glucocorticoids
Source: Genes (Basel). 2026 Sep 24;17(10):1181. doi: 10.3390/genes17101181 (PMC13704200; doi:10.3390/genes17101181)
Supplement: Supplementary file 1 [file genes-17-01181-s001.zip › genes-4506112-supplementary/Supplementary Figures_Genes_v2.pdf]

SUPPLEMENTARY INFORMATION – Figures

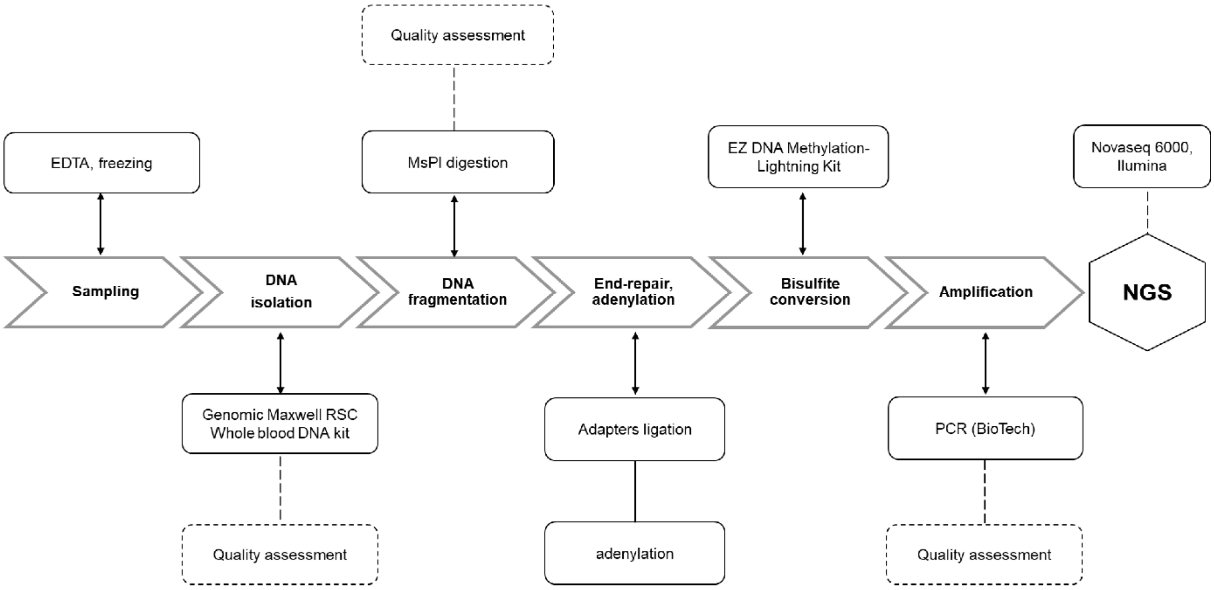

Figure S1

Figure S1. A summary of the laboratory procedures applied in the study.

Abbreviations: MspI – *Moraxella species* I, NGS – next generation sequencing, PCR – polymerase chain reaction, RSC – rapid sample conditioning

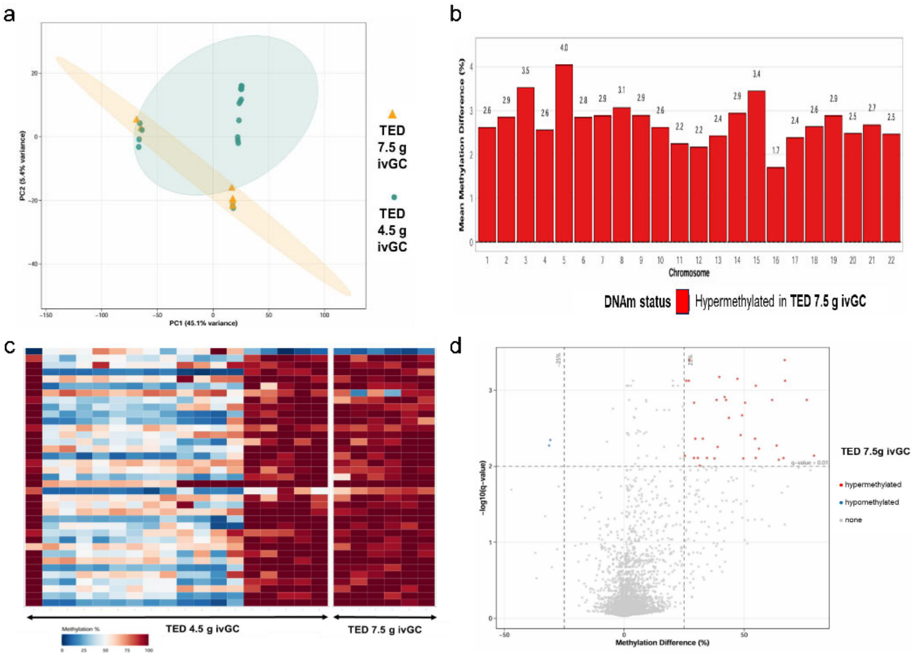

Figure S2a-d

Figure S2a-d. Exploratory analysis according to cumulative intravenous methylprednisolone dose in TED patients. Figure S2a. Principal component analysis plot showing sample grouping of TED 7.5 g ivGC (orange triangles) and TED 4.5 g ivGC (green dots) patients, based on DNAm level of individual CpG. Figure S2b. Absolute DNAm difference stratified by each autosomal chromosome in TED 7.5 g ivGC and TED 4.5 g ivGC patients comparison. Figure S2c. Heatmap presenting clusters of DNAm patterns (rows, top 100 DMCs) in studied subjects (arrows, columns) stratified by total ivGC dose used in treatment; hypomethylation and hypermethylation are expressed by blue and red, respectively; color intensity of the pattern indicates its DNAm level in line with a scale below the chart. Figure S2d. Significant DMCs of TED 7.5 g ivGC vs.TED 4.5 g ivGC patients comparison; q-value 0.01 (a horizontal dashed line). Volcano plot presents differential methylation difference (threshold 25%; vertical dashed lines) on x axis and $-\log_{10}$(q-value) on y axis. Non-grey colored points represent significant hypomethylated (blue; n=2) and hypermethylated (red; n=35) CpG sites, respectively. Distance from center indicates effect size magnitude, DMCs of highest rank are located at the top of the figure.

Abbreviations: DMC – differentially methylated cytosine, DNAm – DNA methylation, log – logarithm, PC1 – principal component 1., PC2 – principal component 2., TED 4.5 g ivGC – nn thyroid eye disease patients after 4.5 g ivGC therapy, TED 7.5 g ivGC – thyroid eye disease patients after 7.5 g ivGC therapy

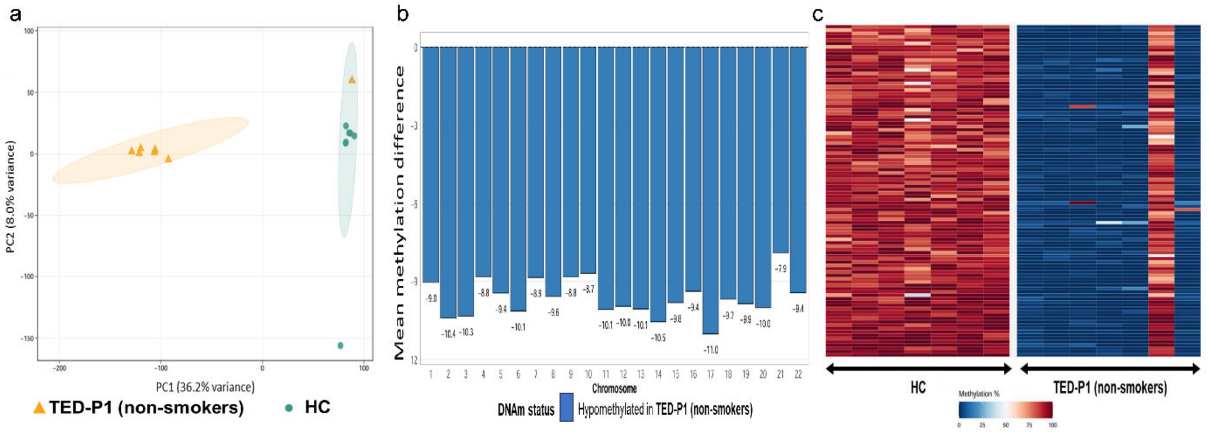

Figure S3a-c

Figure S3a-c. Sensitivity analysis of DNA methylation patterns restricted to never-smokers. Figure S3a. Principal component analysis plot showing sample grouping of never-smoking TED-P1 patients (orange triangles; n = 7) and never-smoking HC (green dots; n = 7), based on DNAm levels of individual CpG sites. Figure S3b. Mean DNAm difference stratified by autosomal chromosome in the comparison between never-smoking TED-P1 patients and HC. Figure S3c. Heatmap presenting DNAm patterns of the top 100 DMCs (rows) across individual never-smoking TED-P1 patients and HC (columns); hypomethylation and hypermethylation are represented by blue and red, respectively, with color intensity indicating the DNAm level according to the scale below the heatmap.

Abbreviations: CpG – cytosine-phosphate-guanine dinucleotide; DMC – differentially methylated cytosine; DNAm – DNA methylation; HC – healthy controls; PC1 – principal component 1; PC2 – principal component 2; TED-P1 – thyroid eye disease patients before intravenous glucocorticoid therapy.

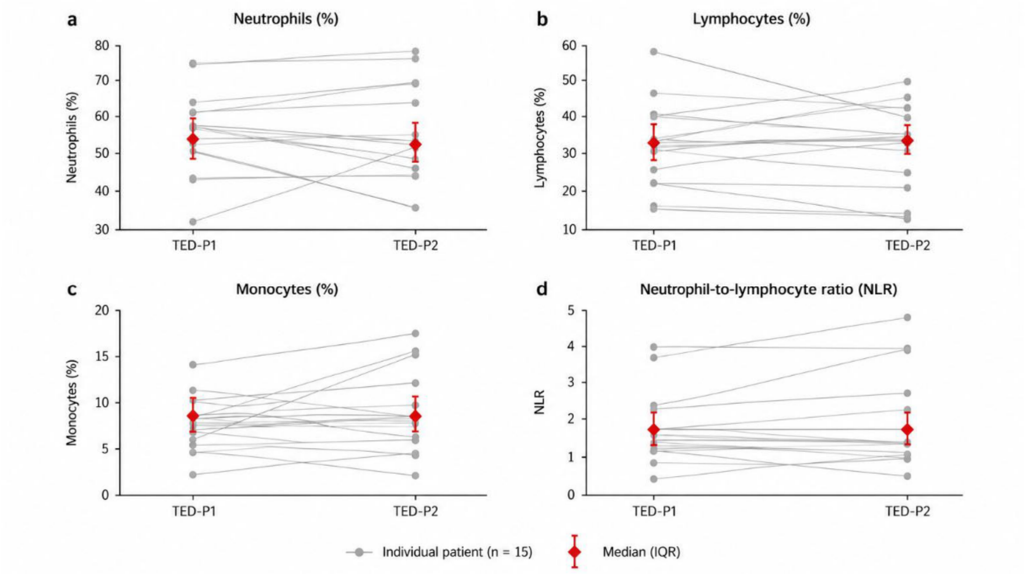

Figure S4a-d

Figure S4a-d. Paired analysis of peripheral blood-cell composition in TED patients before and after intravenous glucocorticoid therapy. Figure S4a. Neutrophil percentage. Figure S4b. Lymphocyte percentage. Figure S4c. Monocyte percentage. Figure S4d. Neutrophil-to-lymphocyte ratio (NLR). Paired measurements are shown for 15 patients with available complete blood counts at both TED-P1 and TED-P2. Grey points represent individual measurements and grey lines connect paired samples from the same patient. Red diamonds and error bars indicate the median and interquartile range (IQR), respectively.

Abbreviations: IQR – interquartile range; NLR – neutrophil-to-lymphocyte ratio; TED-P1 – thyroid eye disease patients before intravenous glucocorticoid therapy; TED-P2 – thyroid eye disease patients after intravenous glucocorticoid therapy.
